# Experiment protocols for brain-body imaging of locomotion: A systematic review

**DOI:** 10.3389/fnins.2023.1051500

**Published:** 2023-03-01

**Authors:** Soroush Korivand, Nader Jalili, Jiaqi Gong

**Affiliations:** ^1^Department of Mechanical Engineering, The University of Alabama, Tuscaloosa, AL, United States; ^2^Department of Computer Science, The University of Alabama, Tuscaloosa, AL, United States

**Keywords:** human locomotion, observational constraints, brain body imaging, experimental design methodology, cognitive demands, locomotive task, brain muscle functional connectivity

## Abstract

**Introduction:**

Human locomotion is affected by several factors, such as growth and aging, health conditions, and physical activity levels for maintaining overall health and well-being. Notably, impaired locomotion is a prevalent cause of disability, significantly impacting the quality of life of individuals. The uniqueness and high prevalence of human locomotion have led to a surge of research to develop experimental protocols for studying the brain substrates, muscle responses, and motion signatures associated with locomotion. However, from a technical perspective, reproducing locomotion experiments has been challenging due to the lack of standardized protocols and benchmarking tools, which impairs the evaluation of research quality and the validation of previous findings.

**Methods:**

This paper addresses the challenges by conducting a systematic review of existing neuroimaging studies on human locomotion, focusing on the settings of experimental protocols, such as locomotion intensity, duration, distance, adopted brain imaging technologies, and corresponding brain activation patterns. Also, this study provides practical recommendations for future experiment protocols.

**Results:**

The findings indicate that EEG is the preferred neuroimaging sensor for detecting brain activity patterns, compared to fMRI, fNIRS, and PET. Walking is the most studied human locomotion task, likely due to its fundamental nature and status as a reference task. In contrast, running has received little attention in research. Additionally, cycling on an ergometer at a speed of 60 rpm using fNIRS has provided some research basis. Dual-task walking tasks are typically used to observe changes in cognitive function. Moreover, research on locomotion has primarily focused on healthy individuals, as this is the scenario most closely resembling free-living activity in real-world environments.

**Discussion:**

Finally, the paper outlines the standards and recommendations for setting up future experiment protocols based on the review findings. It discusses the impact of neurological and musculoskeletal factors, as well as the cognitive and locomotive demands, on the experiment design. It also considers the limitations imposed by the sensing techniques used, including the acceptable level of motion artifacts in brain-body imaging experiments and the effects of spatial and temporal resolutions on brain sensor performance. Additionally, various experiment protocol constraints that need to be addressed and analyzed are explained.

## 1. Introduction

About 28% of American adults older than 50 in the general community presented with impaired locomotion and its prevalence increased with age (*p* < 0.001) (Mahlknecht et al., 2013). The causes of impaired locomotion are divided into neurological (e.g., Parkinson's disease, stroke, Multiple Sclerosis, and dementia) (Allali et al., 2018; Buckley et al., 2019) and/or musculoskeletal drivers, such as arthritis and cardiovascular conditions (Blyth et al., 2019; Andonian and Huffman, 2020; Minetto et al., 2020). Among these neurological and musculoskeletal impacts, previous research has identified different patterns of gait disorders, such as parkinsonian (De Bartolo et al., 2020; Guayacán and Mart́ınez, 2021), frontal (Hülser et al., 2022), or spastic gait (Muñoz-Lasa et al., 2019; Norbye et al., 2020). Furthermore, researchers have argued that locomotion should be defined as a syndrome for pre-clinical outcomes, such as motoric cognitive risk syndrome for pre-dementia (Xiang et al., 2021; Li et al., 2022a). Because of the neurological and musculoskeletal correlates of locomotion, rehabilitation treatments have been explored to utilize locomotion training, such as treadmill gait exercise, to improve patients' musculoskeletal ability and further induce neurological benefits (Smania et al., 2011; Hornby et al., 2016; Bassiri et al., 2022). Therefore, each human locomotion contains unique features of neural and musculoskeletal drivers, clinical conditions, the complexities of human development and aging, and signifiers of physical activity for health and wellness (Runge and Hunter, 2006; Pons et al., 2013; Kerkman et al., 2018).

Understanding the neurological and musculoskeletal correlates of locomotion is a pivotal need to pave the way for successful rehabilitation, improve performing locomotion tasks, especially in older adults, or create a digital twin of humans while doing a locomotion task (Dai et al., 2022). Various techniques, such as electroencephalography (EEG), functional near-infrared spectroscopy (fNIRS), and magnetic resonance imaging (MRI), have been developed and deployed to study age-related changes and specific diseases in the neurological and musculoskeletal correlates (Lewis et al., 2019). These various techniques enable us access to the movement phenotypes, such as brain structures (Chenausky and Tager-Flusberg, 2022), the functional substrates (Magrinelli et al., 2021), and motion signatures (Klibaite et al., 2022), in locomotion control during different experimental settings. However, these neuroimaging techniques present advantages and limitations, such as sensitivity to motion artifacts (Abtahi et al., 2020; Bonnal et al., 2022), portability (Sejdić et al., 2019), and spatial and temporal resolutions (Martini et al., 2020; Kumar and Michmizos, 2022). These limitations set up constraints on the design of the experimental protocols. For instance, MRI studies mainly investigated imagined locomotion rather than real-world locomotion (Stolbkov et al., 2019; Skinner et al., 2022). Although the sensitivity of fNIRS and EEG to artifacts has been improved, still most existing studies focus on low-intensity movement (e.g., walking) rather than high intensity, such as running.

To overcome these challenges and limitations of traditional brain imaging techniques, most recent research argued that brain-body imaging techniques that enable simultaneous measurements of dynamics of brain activities and body movements could be a promising method to reveal the profound relationship between brain, body, and behavior (Makeig et al., 2009; Gramann et al., 2010; Gwin et al., 2010; Wagner et al., 2012). The brain/body imaging techniques integrated portable devices, reliable sensing methods against motion artifacts, and capable of monitoring brain activities and locomotion with appropriate temporal and spatial resolutions, and sophisticated data analysis approaches for multi-modal data preprocessing and curation. Thus far, some specialized centers and clinics such as GE HealthCare (HealthCare, 2023), SCL Health (Group, 2023), Stratus (Stratus, 2023), Zeto (Zeto, 2023), CMS (CMS, 2023), CNS (Calyx, 2023), NordicNeuroLab (NordicNeuroLab, 2023), and NIRx (NIRx, 2023) have adopted these brain/body imaging tools. With multiple-channel EEG (more than 64) capturing brain activities, these tools track spatial information of brain correlates of human motion and provide high-precision data at a sampling rate of 200–5,00 Hz (Cortney Bradford et al., 2019). Simultaneously, they utilized standard locomotion tools, such as optical motion capture systems (Divya and Peter, 2022), inertial measurement units (Khaksar et al., 2021), and EMG sensors (Hallett et al., 2021) to capture the locomotion data at the same sampling rate as brain activity data. As a result, sophisticated data analysis approaches have been developed to study the relationship between brain, body, and behavior, such as cognitive-motor interference and coherence (Zhu et al., 2022).

However, before these brain-body imaging tools can truly be adopted for clinical use, their effectiveness must be carefully assessed. From a technical point of view, reproducing human locomotion experiments has been problematic due to the lack of protocols, standardization, and benchmarking tools, which ultimately impairs the evaluation of previous research quality and validation of prior understandings (Parmentier et al., 2020; Kameli et al., 2021). Notably, only a few studies focused on developing standard protocols and related design methodology as an open research issue. Therefore, this paper focuses on reviewing the settings of existing experiment protocols, such as locomotion intensity, duration, distance, brain sensor technologies, and corresponding brain activation expressions. First, we develop a conceptual framework to identify the design methodology of experiment protocols. Limitations of each brain-body imaging technique and the corresponding constraints on protocol design are then characterized and mapped into the scope of the systematic review approach. Next, we review existing studies that implement various types of experiment protocols, demonstrating the current gaps in design methodology. Finally, metrics for evaluating the research quality and implications for reproducing prior knowledge are proposed.

Overall, we aim to provide a roadmap for the future development of locomotion analysis methods based on brain-body imaging techniques, including highlighting current progress, identifying various constraints, and suggesting potential research directions. The main contributions of this review paper are to:

- Establish intrinsic links between neurological and musculoskeletal correlates of locomotion characteristics and quantifiable measures that brain-body imaging tools can capture;
- Review existing experimental protocols for studying neurological and musculoskeletal correlates of locomotion;
- Examine the feasibility of replicating the experiments in the laboratory systems, and finally,
- Identify gaps and lay out a roadmap for the design methodology of experiment protocols.

## 2. Methodology

**Figure 1:**
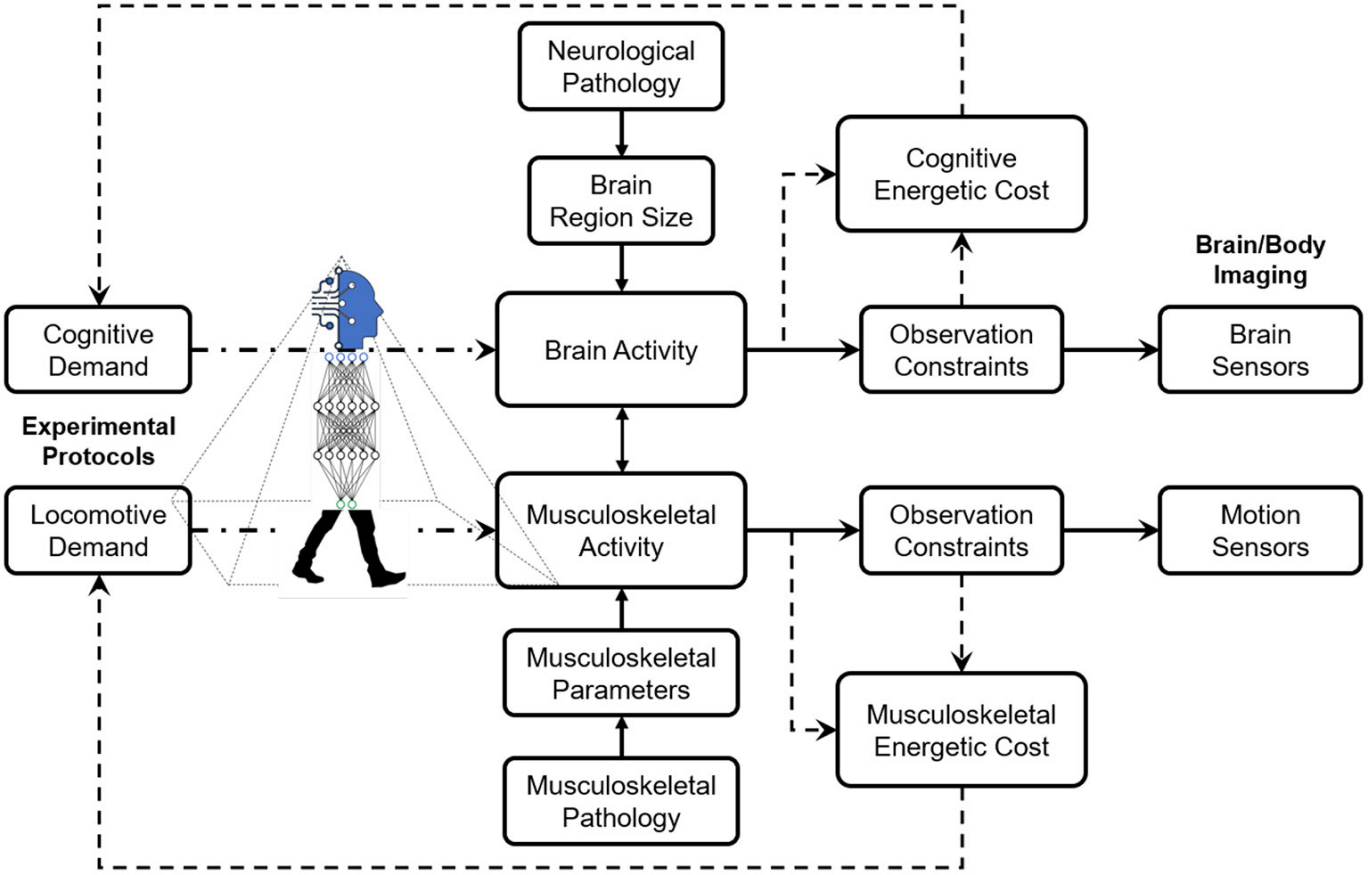
The design methodology of experimental protocols is based on comprehensive considerations of cognitive and locomotive demands, observation constraints by the sensing technology, and energetic costs determined by the human subjects.

The design methodology of experimental protocols for studies on neurological and musculoskeletal correlates of human locomotion is based on careful considerations of cognitive and locomotive demands, observation constrained by the sensing technology, the pathology derived from the research interests, and energetic costs invested by the human subjects. Ideally, the cognitive and locomotive demands are designed in specific thresholds to stimulate certain intertwined relationships between neurological and/or musculoskeletal activities. However, the sensing technology's thresholds of these demands are constrained, including sensitivity to motion artifacts, portability in different environments, and spatial and temporal resolutions for capturing dynamics. For instance, current sensing techniques, such as EEG and fNIRS, claim they have lowered their sensitivity to motion artifacts. Still, most researchers design low-intensity locomotion (e.g., slow walking) to avoid difficulty in denoising efforts. Sometimes, in high-intensity locomotion experiments (e.g., running), the data become useless due to a high amount of movement artifacts (Gwin et al., 2011). Figure 1 illustrates the conceptual framework of the design methodology of experimental protocols.

**Figure 2:**
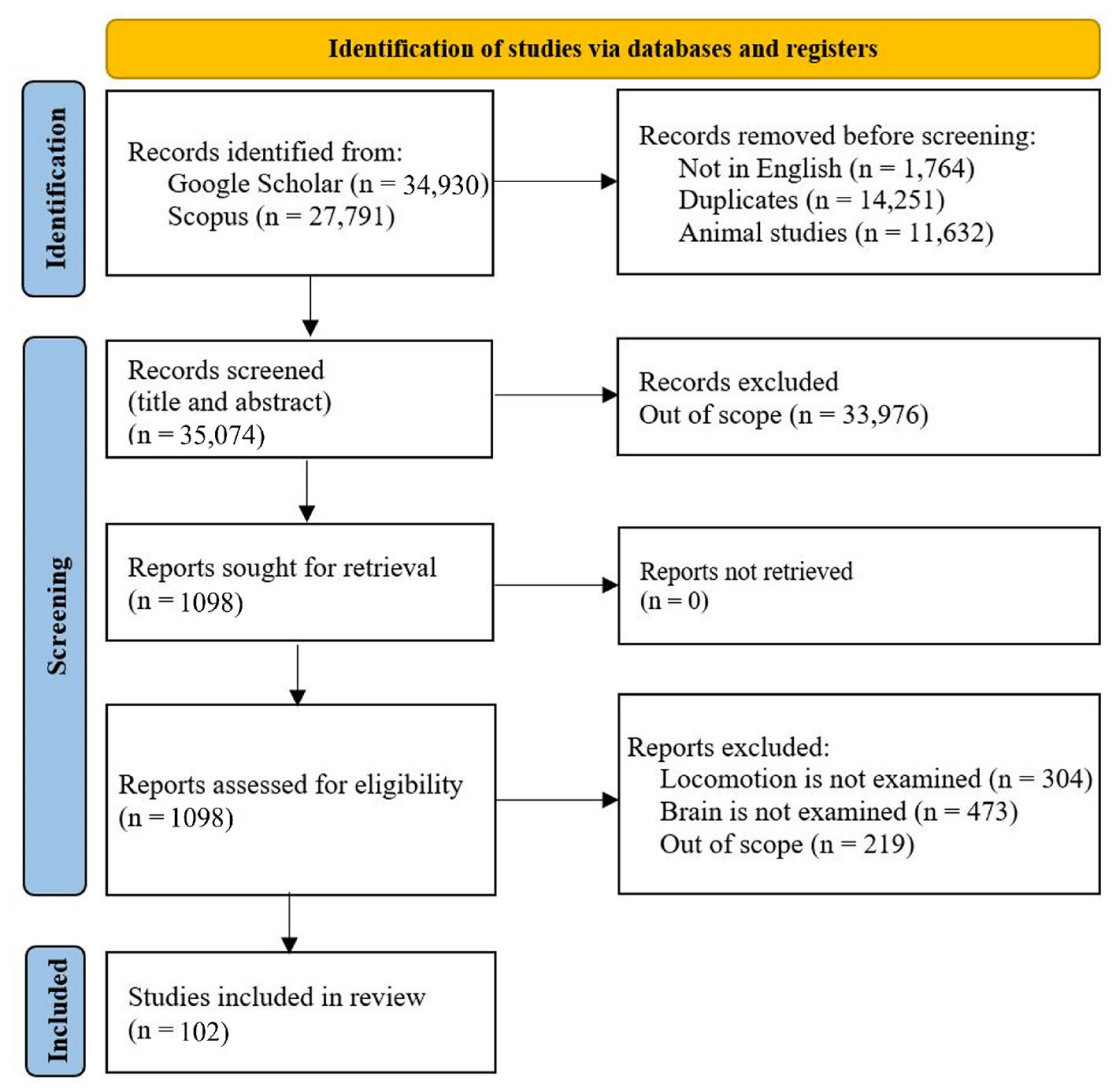
Mobile body imaging study selection: Preferred items for systematic reviews and meta-analyzes (PRISMA) flow diagram.

We developed a systematic search of existing studies based on the conceptual framework. To find the papers, these keywords were searched for in Google Scholar: human brain locomotion/locomotion, OR EEG, OR nirs, OR MRI/ Brain-Body Imaging. Then, all the papers were examined, and the related papers were added to the tables of this paper. The criteria for a paper to be reviewed are: 1) it must examine the brain's signals while the participants perform a locomotion task. 2) The experiment participants should be healthy; the results of this systematic search are shown in Figure 2. Although exploring walking disorders is highly valuable, there are thorough reviews for each locomotion disease. Then, the critical information on protocol design has been extracted and inserted into the tables. In this respect, these parameters in each protocol have been extracted: the type of the surface or the device used for experimenting (e.g., overground and treadmill), the speed of performing the task, the distance that participants have moved, the duration of the task, the type of the sensors used in the experiment (it is more focused on the sensors to read the brain's data), number and age of the participants, special conditions of each research, and the contribution of the research.

All the results were converted to the same units to make the protocols comparable. Accordingly, the distance is stated in meters (m), the speed is in kilometers per hour (km/h), and the duration of the task is in minutes (min); otherwise, the unit is stated.

## 3. Locomotive and cognitive demands

To examine neural activities during human locomotion, single tasks without cognitive demands (e.g., walking) and dual tasks with cognitive demands (e.g., talking while walking) are examined. In this paper, the tasks are divided into walking, running, cycling, and Dual-Task-Walking (DTW). The abbreviations used in this paper are explained in Table 1.

- In the participant section, M shows the number of male participants and the rest are Females.
- a ± b is used to describe the participants' age statistics in which a is mean and b is standard deviation.
- If participants are older than 60, they are considered as OA.

**Table 1:** Abbreviation table.

**Abbreviation**	**Term**	**Abbreviation**	**Term**
B	Board with pedals	deoxyHb	De-Oxygenated Hemoglobin
EEG	Electroencephalogram	EMG	Electromyography
Erg	Ergometer	IM	Imaginary locomotion
M1	Primary Motor Cortex	min	Minute
MRI	Magnetic Resonance Imaging	NIRS	Near-Infrared Spectroscopy
OA	Older Adults	OG	Overground
oxyHb	Oxygenated Hemoglobin	PET	Positron Emission Tomography
PFC	Pre-Frontal Cortex	PMC	Pre-Motor Cortex
rpm	Revolution Per Min	s	Second(s)
S1	Primary Sensorimotor Cortex	SMA	Supplementary Motor Area
SMC	Sensorimotor Cortex	SVM	Support Vector Machine
SW	Single Walking	T	Tesla
Tr	Treadmill	YA	Younger Adults

### 3.1. Type 1: Walking

Walking has been examined in different forms: actual walking, imaginary walking, and simulated walking. Nonetheless, the common point between these studies is the activation of the brain and involvement of higher cognition control areas (Al-Yahya et al., 2011). The studies that have investigated the walking as the human locomotion are shown in Table 2.

**Table 2:** Brain-body imaging in walking.

**References**	**Surface**	**Dist. (m)**	**Speed Km/h**	**Duration (min)**	**Sensors**	**Participants No. and age**	**Special condition**	**Contribution**
Vitorio et al. (2018)	Treadmill	-	Self-paced	5 min: 5 trials of 30 s SW and 30 s rhythmic auditory cueing walking	40-channel fNIRS	15 YA Range: 20–40, 15 OA Older than 60	A digital metronome was used to create rhythmic auditory cueing with preferred frequency	In OA, rhythmic auditory cueing enhances walking, which is achieved through increased activity in multiple cortical areas (PMC, SMA, and M1). In OA, cortical response reduction with repeated exposure indicates OA's ability to adapt to a new task.
Kurz et al. (2012)	Treadmill	-	1.62	5 min: five blocks consisting of 30 s standing still and 30 s walking	24-channel fNIRS	13, 23.7 ± 1.4	Forward or backward walking condition is placed in five alternating blocks of standing still or walking. Backward walking needs more SMC and increases the variability of the stride time compared to forward walking.	Amount of oxyHb during forward walking is correlated with the variability of stride time in forward walking in the pre-central gyrus and SMA of the brain.
Khan et al. (2018)	Treadmill	-	Self-paced	10 s walking and 20 s for rest intervals	12-channel fNIRS, 4 detector and 5 sources	9M (30 ± 3)	The fNIRS data were collected from M1 only on the left hemisphere	Initiation and stopping commands received from M1 in the left hemisphere obtained by fNIRS are classified with different ML algorithms.
Berger et al. (2019)	Treadmill	-	2.8	One block of each walking condition consisted of 5 trials and each trail lasts for 1 min with 1 min interval.	fNIRS with 16 optodes (8 × 8) and ground reaction forces	12 right-handed (3M), 25 ± 4	Two conditions of unassisted walking and robot assisted walking are compared. Body weight support is adjusted to 30%.	Using robot assisted walking causes an increase in gait variability, which is correlated with increasing brain activity in SMC.
Suzuki et al. (2008)	Treadmill	-	3	Two walking tasks and total duration of each task was 240 s	fNIRS (42 channels) with 28 optodes, consisting of 12 light-source fibers and 16 detectors	7 right-handed (4M), 31.3 ± 4.8	Duration of rest period was selected randomly (10, 15, 20, and 25). The walking tasks were repeated 4 times.	When walking triggers with a verbal instruction (“ready”), the brain's frontal activation during the preparation, execution, and walking performance improves.
Miyai et al. (2001)	Treadmill	-	1	Each task or rest period lasted 32 s (8 scans). each series contained 80 scans with a complete duration of 5 min 20 s.	NIRS (21 optodes), which consist of nine light source fibers and 12 detectors	8 (4M), 35 ± 8, Range: 24–46	Performing each of these tasks for 30 s (1) walking on the treadmill at 1.0 km/h and rest, (2) alternating arm swing without walking and rest in standing position, (3) alternating dorsiflexion and plantar flexion movement of the feet at a pace of 1 Hz and rest in sitting position, and (4) motor imagery of gait in standing position	Using local oxyHb measured by NIRS topography, cortical activation patterns of human gait is visualized. Total and increased level of oxygenated hemoglobin in the medial S1 and SMA are coupled with walking activities.
Harada et al. (2009)	Treadmill	-	High gait capacity: 3.6 ± 1, 5.8 ± 1, 6.9 ± 1 Low gait capacity: 3.1 ± 1, 4.4 ± 1, 5.2 ± 0.7	20 s rest, 60 s walking, 20 s rest	NIRS (42 channel) with 28 optodes consisting of 12 light sources and 16 detectors	15 (2M), 63 ± 4	Dividing the participants to two groups based on incremental walking test: 1- low gait capacity (*n* = 8) 2-high gait capacity (*n* = 7)	In higher speeds, left PFC and the SMA consists of higher oxyHb. The degree of medial SMC and SMA activations are associated with the locomotor speed and cadence. Heart rate is only depended on left PFC. Left PFC, SMA, and SMC control gait speed, and that the involvement of the left PFC may rely on reduction of gait capacity in elder adults.
Sipp et al. (2013)	Treadmill (customized by adding a beam to evaluate the stability)	-	0.792	25 min, two times	EEG (256 channels), Vicon motion capture with 28 reflective markers	26 (14M), 23 ± 5, All of them right-handed and right footed	To avoid the effect of fatigue on the experiment, participants could walk whenever they wanted. Accordingly, the time of experiment could last between 60 to 120 min.	Walking involves baseline theta band activity that significantly increases with loss of balance. Also, in the right-handed and footed participants, the left SMC plays a larger role in sensing loss of balance during walking than the right SMC.
Gwin et al. (2011)	Treadmill	-	2.88 (slow walking) and 4.5 (fast walking)	5 min standing 10 min slow walking 10 min fast walking	EEG (248 channel), force measuring on the treadmill with 25 reflective markers	8 (1M) range: 21–31	The experiments start with 5 min standing followed by three random conditions: slow walking, fast walking, or running. Collected data from running was not used because of large mechanical artifacts	Alpha and beta band spectral power increase around sensorimotor and dorsal anterior cingulate cortex at the end of the stance phase. But, their increase around the sensorimotor during the push off is more noticeable. Changes in anterior cingulate, posterior parietal, and SMC were pronounced in intra-stride high-gamma spectral power.
Bruijn et al. (2015)	Treadmill (split-belt)	-	3.6	30 min	EEG (64 channel), ground force sensors, 2 pairs of bipolar EMG, cluster-markers and Optotrack	10 (7M), 31.4 ± 6.6	The experiment consists 5 conditions: Sitting with open eyes (1 min), sitting while receiving stimuli to the medial nerve (7 min), normal walking on treadmill (10 min), supported walking on treadmill (10 min), walking while receiving stimuli (10 min)	During stabilized walking, beta band increases in the left M1 indicating the role of the part in steady state gait stability. Confirming that medio-lateral foot placement is determined during push-off to some degree.
Bradford et al. (2016)	Treadmill	-	2.7	30 min walk at 0% grade	EEG (264 channel), EMG (6 channel), ground forces	22 (12M), 23.1 ± 3.9	Subjects walked for a total of 1 h at 0.75 m/s. Subjects alternately walked in 7.5-min blocks of time at 0% grade and at 15% grade, for a total of 30 min at each condition	Comparison of walking with 0% and 15% grades shows greater gamma power during level walking in the left sensorimotor and anterior cingulate clusters. Also, comparison of frequency activation of the artifacts during walking conditions shows that the differences between walking conditions were cortically driven rather than a residual artifact of the experiment.
Salazar-Varas et al. (2015)	Treadmill	-	2	12 min combination of different reactions to the appeared obstacles.	EEG (32 channel), 7 IMU	5M, range: 24–29	To create obstacles, in one scenario a line laser is projected over the treadmill to simulate the appearance of the obstacle. In the second scenario, a screen placed in front of the treadmill changes its color to simulate the appearance of the obstacle	EEG role can be developed to detect the unexpected obstacles as EEG potential over the fronto-central area of the subjects' brain change. An accuracy of 79.5% was reported for obstacle detection.
Bulea et al. (2015)	Treadmill	-	3.24 (slow walking) 5.4 (fast waking) and self- adjusted speed	1 min data collection by EEG cap in two modes of passive and active.	EEG (64 channels), Vicon MX motion capture system	10 (4M) 28.9 ± 6.3	The experiment consists of passive walking (defined speed) in which the speed periodically changes. Or active walking (self-adjusted). In the active mode, combination of feedforward and feedback controllers were implemented. Each participant completed 6 trials	Gamma band power increases during double support and early swing phases. This indicates that pre-frontal and posterior parietal networks are engaged to leverage lower limb control during walking. The cortical network engagement evoked by active treadmill indicates the possibility of enhancing neuroplasticity for more effective motor training.
Yokoyama et al. (2018)	Treadmill	-	2	7 min and 30 s	64-channel EEG and 13-channel EMG also 3D ground reaction forces	12M, Range: 23–31	The last 7 min of walking and 30 channels' data of EEG cap were used for analysis	During walking cerebral cortex controls multiple muscles hierarchically through a few muscle synergies. Locomotor muscle synergies activation can be decoded from slow cortical waves.
Presacco et al. (2011)	Treadmill	-	Self-paced	5 min to find the comfort speed, 2 min rest, 5 min precise walking, then normal walking	EEG (60 channel), EMG, infrared optical motion capture system	6 (3M), Rang: 18–45	To increase attentional demand during precision walking condition, participants were instructed to avoid stepping on the white stripe (2 in. wide) glued diagonally on the treadmill's belt by using the monitor's video to keep track of foot placement relative to the white stripe	Confirming that a plurality of cortical brain areas controls the walking. Decoding human walking using EEG data in two conditions: 1- walking while providing foot placement guide visually (precise walking) 2- normal walking
Presacco et al. (2012)	Treadmill	-	Self-paced	5 min to find the comfort speed, 2 min rest, 5 min precise walking, then normal walking	EEG (60 channel), EMG, infrared optical motion capture system	6 (3M), Rang: 18–45	To increase attentional demand during precision walking condition, participants were instructed to avoid stepping on the white stripe (2 in. wide) glued diagonally on the treadmill's belt by using the monitor's video to keep track of foot placement relative to the white stripe	Activation of ankle, knee, and hip during walking on treadmill are decoded by recording 12 EEG signal channel placed on pre-frontal, motor, parietal, and occipital areas.
Castermans et al. (2014)	Treadmill	-	1.5, 3, and 4.5	About 12 min	EEG (32 channel), piezoelectric accelerometer, 6 infrared cameras to record lower limb movements	7 (5M), Range: 25–33	The piezoelectric accelerometer was fixed firmly on top of the participants' head	The role of cortical origin of low-delta and high-gamma bands during walking may not be valid. Depending on the electrodes' locations, motion artifacts in phase with walking frequency can affect the EEG data up to 15 Hz. Accelerometer and EEG data have similar time-frequency characteristics during walking.
Petersen et al. (2012)	Treadmill	-	Self-paced between 3.5 to 4	5 min epochs of continuous treadmill walking and 2 min of static contraction	EEG (28 electrode), EMG	9 (4M), 23.4 ± 4.1	additional walking with speed of 1 km/h (slow walking) and static dorsiflexion for seven of the participants	Rhythmic cortical activity in the 24–40 Hz frequency band is transmitted *via* the corticospinal tract to the active muscles. In steady state treadmill walking, motor cortex and corticospinal tract contribute directly to the muscle activities.
Quiroz et al. (2017)	Treadmill	-	4.83 and 8.05	5 min	EEG (9 channels)	3	Each participant repeated the experiment 10 times: 1 min low speed, 1 min higher speed, then 1 min low speed also 1 min between transitions	Compared to imager locomotion, during lower limb control, neural activity in cortical sensorimotor areas increases. Also, pre-motor and sensorimotor areas' show high neural activities compared to resting.
Artoni et al. (2017)	Treadmill	-	2.5 and 3.5	20 min (10 min for each speed) and also 5-min rest between the tasks	EEG (64 Channels), EMG (6 channels)	11, 30 ± 4	3-min preliminary walking was performed for the purpose of acclimation	A significant casual unidirectional drive from contralateral motor cortex to muscles in the swing leg and control of muscles during stereotyped treadmill locomotion is found using SVM. Highest accuracy was reported as 0.78 ± 0.04.
Tortora et al. (2020)	Treadmill	-	2.5 and 3.5	20 min (10 min for each speed) and also 5-min rest between the tasks	EEG (64 Channels), EMG (6 channels)	11, 30 ± 4	3-min preliminary walking was performed for the purpose of acclimation. A specific set of channels was removed for each of the 11 participants.	Decoding swing and stance of both legs together, or of each leg independently by deep learning and using the method of LSTM recurrent and EEG signals of motor cortex. An average accuracy of 90.4 ± 1.4% is reported.
Wei et al. (2021)	Treadmill	-	1.4, 2 and 2.6	7.5 min: walking at three speed in 15 30-s blocks	24-channel EEG, 8-channel EMG, motion capture,	9 (7M), Range: 23–26	To divide the gait cycle, 3D markers in five positions were employed	Activation of cerebral cortex during gait phases is examined. During pre-swing and terminal-stance, cerebral cortex is more actively involved in the control of eight examined muscles.
Nordin et al. (2019a)	Treadmill	-	1.8, 3.6, 5.4, 7.2	3 min for each speed and considering rest between each experiment	Dual layer EEG and 8-channel EMG	9 (6M), 27 ± 4	As the speed is close to running, the subjects were asked to walk even in high speeds. A standing baseline trial was recorded prior to changing speed experiments.	Dual-layer EEG isolates the changes in sensorimotor electrocortical dynamics across walking speeds. Also, dual-layer EEG is beneficial to remove residual artifacts while gait speeds change. In addition, a correlation between different walking phases and alpha/beta spectral power is drawn.
Luu et al. (2017b)	Treadmill	-	1.6	2 min rest, 15 min Gonio-ctrl walking, 5 min BCI-ctrl walking, 2 min rest	64-channel EEG	8 (3M), Range: 19–29	Goni-ctrl: the avatar was driven by a goniometer. goniometer sensor placed at hip, knee, and ankle joint angles. BCI-ctrl: the avatar was controlled by BCI. The participants could see the avatar on the 52-inch TV.	In closed-loop walking using BCI and avatar, cortical involvement during walking increases as α/μ are subdued in the posterior parietal cortex and inferior parietal lobe. Low γ modulations in the anterior cingulate cortex and superior temporal gurus may show the increasing voluntary control of human gait.
Lin et al. (2014)	Treadmill	-	1.6, 3.2, 4.8	Less than 30 min	14-channel EEG	17 (14M), Range: 22–32 Mean age: 26.76	Each subject participated in four sessions (standing and three speeds). Each session repeated a run 10 times analysis.	The centro-parietal was not covered with the sparse 14-channel electrodes and no spectral changes were observed in SMC. steady-state visual-evoked potential-based BCI can be used to mimic natural walking using consumer-level EEG.
Úbeda et al. (2014)	Treadmill	-	2,3, and 4	24 min	32-channel EEG, IMU	3M, 26.3 ± 3.8 Range: 22–29	Each participant performed 8 runs. Each run includes walking at three speeds and each for 1 min	Using the linear regression model, a correlation between EEG signal recorded from central and parietal cortex and knee angle during walking has been found
Severens et al. (2012)	Treadmill	-	2.8 ± 0.2	10 min	62-channel EEG, EMG channel, occipital channel	6, 21.6 ± 2.3 Range: 20- 26	To help the subject to synchronize the step frequency, a metronome was used for 15 s. The step frequency was about 1.4 Hz.	ERD has been examined and measured during walking. Beta ERD is strongest above the lateral motor cortex, with mu at the central motor cortex. Also, desynchronization is strongest in the swing phase of the contralateral leg above the motor cortex.
Wagner et al. (2016)	Treadmill	12 trials of 10 blocks of 120 steps	Self-paced between 3.0 and 3.7	-	108-channel EEG, EMG channel	18 (10M), 29.1 ± 2.7 (Wagner et al., 2019a), Range: 22–35	the walking was synchronized with a series of cue pacing cue tones and thus the step rate and length were changed based on the pacing cue tempo	Analyzing beta band power in the right dorsolateral prefrontal cortex shows two recognizable patterns. One pattern may help in starting and executing the movement and the other one has control and inhibition functions (Wagner et al., 2016). Also, in the posterior medial frontal cortex, an EEG step-cue delay negatively is generated with a peak at 250 ms after anomalous cue tone onsets (Wagner et al., 2019b).
De Sanctis et al. (2023)	Treadmill	-	Self-paced	28 min (eight 3.5-min blocks)	160-channel EEG, optitrack motion capture with 9 cameras	26 (12M), 74.9	High and low cognitive impairment risk is defined by Montreal Cognitive Assessment battery (MoCA, range: 0- 30): high (22–26) and low (27+)	Characterizing the neural signature of walking: an increase in frontomedial theta in high-risk cognitive impairment individuals was observed. Left sensorimotor beta in low-risk cognitive impairment individuals decreases when visual perturbation is used during walking.
Wagner et al. (2012)	Treadmill/ robotic gait orthosis	-	Speed = 0.54 (leg length in cm)/ 27.8 The speed varied between 1.8 to 2.2	6 min for walking session; 3 min resting session	Combining four 32-channel amplifiers for recording data from 120 sites using EEG and EMG data	14 (8M), 24.3 ± 2.7, Range: 22–28	Each participant completed 8 runs of robot-assisted walking (four in each of two active/passive walking conditions) and three runs of upright standing.	To compensate for the differences between active and PW in robot-assisted walking, cortical activities related to lower limb movements were shown. Depending on the gait cycle, the power in the mu and beta bands decreases during active walking. Also, depending on the gait cycle, cortical activity was localized in the M1 in the lower gamma band. (passive walking: participants let the robot move their legs)
Seeber et al. (2015)	Treadmill/ robotic gait orthosis	-	Speed = 0.54(leg length in cm)/ 27.8 The speed varied between 1.8 and 2.2	6 min for the walking session (4 times); 3 min standing (3 times)	Combining four 32-channel amplifiers for recording data from 120 sites using EEG and EMG data	10 (5M), 25.6 ± 3.5, Range: 22–28	Each participant completed 4 runs of robot-assisted walking (each 6 min), 3 runs of standing (each 3 min)	When high gamma oscillations are increased artificially by transcranial alternating current stimulation (tACS) in the central sensorimotor cortex, motor performance during walking enhances.
Seeber et al. (2014)	Treadmill/ robotic gait orthosis	-	Depending on the leg length, ranging from 1.8 to 2.2	4 runs (6 min each) of active walking and 3 runs of upright standing (3 min each).	EEG (120 channel), EMG	10 (5M), 25.6 ± 3.5	Participants completed Body weight support adjusted to less than 30%	μ (10–12 Hz) and β (18–30 Hz) oscillations in active walking is significantly less than upright standing. Depending on the gait phase, supported μ and β ERD indicate a movement-related state change of cortical excitability. While generated frequencies in μ and β have overlaps, the center of generations is different.
Wagner et al. (2014)	Treadmill/ robotic gait orthosis	-	Speed = 0.54 (leg length in cm)/27.8. The speed varied between	Participants walked 4 min in each of the five conditions and walking was repeated two times during the experiment	EEG (61 channel), EOG (3 channel) electrodes by two 32-channel amplifiers	11 (7M), 26 ± 2	Walking in five different conditions: 1-looking at a black mirror 2- looking at white graphical objects 3- watching their mirrored walking 4- 3rd person in Virtual environment 5-1st person in a virtual environment	In conditions that require adjusting the steps based on the visual input, μ, β, and lower γ frequencies in pre-motor and parietal cortices are reduced, which shows these brain areas' activation increase. This activation is higher compared to mirror feedback and a visual attention task, which may indicate additional motor planning and visuomotor processing.
Alchalabi et al. (2019)	Treadmill real walking/ imagining/ observing	-	-	8.5 s for each trial	19-channel EEG and 15 rigid body reflective motion-capture and 12-camera Vicon optoelectronic motion capture system	20 (7M), 23.3 ± 3.93	The participants performed the experiments in three conditions of 1- controlling an avatar in virtual reality 2- imagining the avatar 3- observing the avatar. Each condition consisted of 240 trials.	It is shown that it is feasible to use pre-motor, motor and parietal areas' EEG data to measure the level of embodiment during physically or mentally controlling an avatar's walking.
Severens et al. (2014)	Treadmill/ imaginary	-	3	48 s for the task	62-channel EEG	12, 29 ± 5.6	Four tasks' EED data: forward walking and backward walking in actual and imaginary manners were recorded on a treadmill.	Although walking is automatic, brain signals, especially the cortical area, can be classified to walking and non-walking signals reliably with high speed. Also, actual waking classification has a higher accuracy compared to imaginary walking.
Nojiri and Iwane (2014)	Imaginary	-	-	2 min: 30 s walking, standing, turning left, and turning right	11-channel EEG	1 participant	For imagination, a movie shows three kinds of arrows and stop sign	Providing a method to estimate walking direction using power spectrum density data of motor area's EEG signals.
Malouin et al. (2003)	Imaginary	-	Qualitat-ively: fast and slow	Each subject experienced eight PET scans within a single session that took approximately 2 h	PET. EMG and ECG were recorded for 1 min just before and during each scan. ANOVA to record heart rate	6 right-handed (1M) Range: 41–70, Mean age: 55.9	Brain scanning while participants imagine Standing, initiating gait, walking, walking with obstacles. The results of these conditions were compared to a rest (control) condition	When the cognition demand of the task and the need for processing sensory information increase, the higher brain centers become more engaged.
Iseki et al. (2008)	Imaginary	-	Observa-tion of virtual walking with the speed of 3.2–3.6	Duration of each clip was 5 s, with the frame rate of 29.97 frame/s	3-Tesla MRI with an 8-channel phased-array head coil	16 healthy, right-handed (13M), 34.3 ± 4.6	Tasks: observation of 1- Gait movement 2- normal stepping in a standing position 3- standing still 4- scrambled gait 5- virtual walking 6- scrambled virtual walking	During imagine of walking (first person) or observing other people's walking (third person), the planning center of gait, including SMA and dorsal M1 are activated.
Labriffe et al. (2017)	Imaginary	-	120 step/min	5 min and 42 s	MRI-compatible Korvit simulator and EMG	18 (11M), 27 ± 4.7, Range: 20–40	The experiment has two modes: 1- organized: sequential activation of muscles related to walking 2-chaotic: non-gait-like pattern activation of muscles. Each experiment repeated 9 times.	There is no difference between activation of chaotic and organized patterns of stimulation. Activation pattern of mental imagery and gait-like plantar stimulation are similar especially in SMA-proper bilaterally and right pre-SMA.
Wang et al. (2009)	Imaginary	-	-	3 min and 40 s for each session	fMRI	21 right-handed gender-balanced, 21.5 ± 1.2, Range: 20–25	The experiment includes ten fMRI sessions consisting of three blocks. Walking and stand positions were randomly shown.	In major gait-related task especially at initiation of a gait, SMA is activated. During termination and stepping over an obstacle, a significant visuomotor network is required.
Sacco et al. (2006)	Imaginary	-	90 step/min	12 s for each active condition	fMRI	12 right-handed and right-footed (7M), Range: 20.8–34.9, mean: 27.5	Each participant completed 25 blocks: 13 rest and 12 active conditions	Imagery training expands active bilateral motor areas and reduces visuospatial activation in the posterior right brain.
Ikeda et al. (2016)	Horizontal free walking on a board/ treadmill	-	60 step/min	500 s: treadmill gait for 100 s and 25 s gait-like motion repeated four times	fMRI and EMG for five muscles treadmill gait	8M, 24.0 ± 0.82	The difference between gait-like motion and real walking which is intention for moving lower extremities and biceps femoris.	It is shown that lower-extremity motion simulator by providing gait-like motion incites motor sensation in cerebellum, brainstem, and spinal.
Sahyoun et al. (2004)	Board (a purpose-built wooden apparatus in MRI device)	-	-	12.5 min for a total experiment	3T Varian INOVA MRI, EMG	12 (7M) healthy right-handed. Mean age: 25.4 Range: 20-31	Only one degree of freedom for foot movement is studied: extension-flexion at the ankle joint	Anterior pre-frontal regions are involved in the decision making for moving forward.
Takahiro et al. (2013)	On board	-	1.8 s for each gait	Four reparations of 25-s rest and 25-s gait-like motion	fMRI	1 subject	Three degree of freedom were created on the board for each leg.	The activation of the brain's area in sensory motor is higher in PW compared to active walking due to processing of the unanticipated sensory feedback and not-imaged movement
Wieser et al. (2010)	Board with adjustable tilt angle	-	44 step/min	7 min rest at α=0 and 30 min stepping at α=76 and again 7 min rest at α=0	64-Channel EEG and record of EMG for Four muscles	20 (9M), 28.6 ± 8.3	Task: Stepping (gait -like), the experimental board was vertical (α=0) and then tilted (α=76°)	S1, M1 and SMA mainly control the human's gait. Also, most of the cortical capacity is used for changing the direction between flexion and extension phase.
Xu et al. (2017b)	OG	4.4 m	Qualitat-ively: Low/ medium/ high	About 4.5–6 s	22-channel fNIRS	30, 21 ± 1	To train the algorithm 15 individuals' data was used and 15 individuals' data was used for validation.	Decoding the walking speeds categorized in three speeds based on PFC, M1, frontal eye cortices, and SMA by oxyHB data and SVM algorithm.
Xu et al. (2017a)	OG	4.4 m	Qualitat-ively: Low/ medium/ high	-	22-channel fNIRS	21, 21 ± 1	12 set of data is used for training and 9 for validation	Classification of walking speed based on oxyHB characteristics using SVM.
Lacerenza et al. (2021)	OG	-	Self-paced	20 s standing then 20 s performing the task and 20 s recovery for five times	Single channel fNIRS	3M (age 30, 55, and 50 years)	The task is combination of standing still, forward walking, and backward walking	Time domain fNIRS during freely walking is measured. Diverse cortical response during forward and backward walking can be related to the different motor cortex involvement.
Peters et al. (2020)	OG	10 mactive and 10 m PW	-	51.4 ± 5.2 (Active) to 52.8 ± 3.6 (Passive) seconds	54-channel fNIRS and EMG	14 (7M) 34 ± 8	In PW, participants were instructed to be relax so that the exoskeleton could perform the walking.	Finding the partial activation of parietal cortex during passive robotics exoskeleton gait
Brantley et al. (2016)	OG, and stairs	7.92	self-paced	-	EEG (64 channel), surface EMG (12 electrodes)	1M, 31	The subject has performed 20 trials. Each trial consists of 26ft level ground walking followed by an 8-step staircase (13.4 cm height)	During level ground walking, EEG-led coupling between electrodes and sEMG (tibialis anterior) in the frequency band of (3–5 Hz) indicates the command signal is sent from cortex to peripheral motor neurons. A higher coherence was observed for frequencies less than 2 Hz during stair ascent in which EMG was the leading signal for biceps femoris and gastrocnemius.
Mehra et al. (2021)	OG, and stairs	-	Self-paced	One-min data recording	EEG (60 channels), EOG (4 channels), EMG (6 channels), IMU sensors.	6 (5M)	Each participant completes 20 trials of level ground walking, slope descends, and stair ascends, then 180° return to the starting point and resting.	Decoding the transition of walking conditions 3.0 ± 1.63 s in advance using EOG signals. This decoding is faster than decoding reported by Luu et al. (2017a) using occipital EEG signals.
Luu et al. (2017a)	OG, and stairs	-	Self-paced	One-min data recording	EEG (60 channels), EOG (4 channels), EMG (6 channels), IMU sensors.	6 (5M)	Each participant completes 20 trials of level ground walking, slope descends, and stair ascends, then 180° return to the starting point and resting	Decoding the transition of walking conditions 1.27 s in advance by observing the changes in the cortical dynamics
Velu and de Sa (2013)	OG	1.5	-	-	64-channel EEG data and two EOG electrodes and 8-channel EEG	9 (7M) right-handed subjects, Range: 18–27	The experiment consisted of 60 trials for 6 conditions (standing still, pointing left or right, walking left or right or front).	Walking, pointing, and standing can be classified using EEG data. Spatial and spectral contributions were from areas related to motor planning and mostly from low frequency cortical activity.
Budde et al. (2016)	OG	10	Self-paced	2 min	64-channel EEG and OptoGait-System	12 (6M), Range: 20–28	The experiments had three conditions: 1- normal walking 2- cognitive interface task: press a button based when a high-pitch sound is heard and ignore the low-pitch sound 3- motor interface task: preventing connection of the rings placed on a stick	Doing tasks that involve the brain's motor interface reduces gait velocity and stride length and increases the stride time and temporal-spatial variability. These changes don't occur in tasks require cognitive interface involvement.
Li et al. (2016)	OG	-	Self-paced	-	62-channel EEG 2-channel EOG 4-channel EMG	7M, 23.57 ± 1.51	The experiments were performed in free walking, using exoskeleton with and without applying assistive torque	The activation pattern during walking when the exoskeleton is used and when it is not used is different, which can affect the rehabilitation procedure and further orthosis development. Though somewhat similar in spatial pattern distribution in the medium occipital cortex and parietal cortex, and the lateral temporal cortices, assistive walking shows higher activation in the frontal part compared to two other conditions
Li et al. (2019)	OG	21	Self-paced	-	62-channel EEG, 1-channel EOG, 4-channel EMG	27M, 4 ± 2.32	The participants performed four overground walking: free walking, walking with exoskeleton without applying force and with low and high applied load force	Power spectral density is different in sensorimotor and posterior parietal areas in four different walking conditions. Power spectral density of the brain in conditions of walking while wearing the exoskeleton have more similarities together than free walking.
Nakagome et al. (2017)	OG	-	Self-paced	-	64-channel EEG, 6-channel EMG, 17 IMU	6 (5M)	Level ground walking, stair descent, stair ascent, ramp ascent, and ramp descent are the activities that participants performed. Each participant performed the tasks for 20 times.	Unscented Kalman Filter was used to predict limbs activation using EEG signals.
Weersink et al. (2019)	OG	150	Self-paced	-	32-channel EEG	20 (7M), 64.95 ± 7.2	The participants performed two experiments with 10 min in between: 1- walking normal (with swinging arm) 2-walking without swinging arm.	The relation between arm swing in walking and a step ERD-ERS pattern in high-beta/low-gamma band with the SMA shows the SMA's role in integration of cyclic anti-phase movement of upper and lower limbs.
Hasan et al. (2019)	-	-	Self-paced	1.5 s before the event and 0.5 s of post event	8-channel EEG and one-channel EMG and 9 IMU sensors	7 (5M), 27.4 ± 3.1	Each participant completed 140 trials, which consists of rest, start walking, stop walking and rest again.	Classification of walking intention (active) and non-intention (in-active) by SVM

### 3.2. Type 2: Running

Only one paper was found that merely focused on examining running as a locomotion task and studying brain activities. This paper's information is described in Table 3.

**Table 3:** Brain-body imaging in running.

**References**	**Surface**	**Dist. (m)**	**Speed Km/h**	**Duration (min)**	**Sensors**	**Participants No. and age**	**Special condition**	**Contribution**
Giles et al. (2018)	Treadmill	-	As fast as heart rate is withing 75–85% of age adjusted heart rate	90 min for the main task	20-channel fNIRS	24 (9M) right-handed individuals, Range: 18–33	Emotion regulation were performed before warming up and was reminded every 15 min during running	An emotion regulation strategy is suggested to benefit psychological state during endurance exercise. This benefit may not be reflected in oxygenation of PFC.

### 3.3. Type 3: Cycling

Cycling is another locomotive task to study the brain correlates of locomotion. The locomotive demands span from low to high intensity, with various cycling speeds, while few studies gave self-paced instructions to participants. Most studies expected to utilize the locomotive demands to stimulate the dynamics of brain activities for identifying neurological and musculoskeletal benefits. The research studies related to the cycling are demonstrated in Table 4.

**Table 4:** Brain-body imaging in cycling.

**References**	**Surface**	**Speed Km/h**	**Duration (min)**	**Sensors**	**Participants No. and age**	**Special condition**	**Contribution**
Billaut et al. (2013)	Ergometer cycle	128.7 ± 12.5	Fifteen 5-s cycling sprints interspersed with 25 s of rest	2 pairs of NIRS and EMG from three muscles.	10M athlete 22.8 ± 4.4	All of the participants are from sports clubs and have cycling experience	According to PFC data recorded by fNIRS, during intermittent, short, sprints, central; nervous system regulates quadriceps muscle recruitment and limits the development of muscle fatigue.
Keramidas et al. (2011)	Ergometer cycle	Self-paced (60–90)	30 min for the constant power testing	3-pairs of NIRS	8, 23.9 ± 4.6	The experiments consisted of three parts: 1- maximal oxygen uptake 2- a control constant power test 3- a constant power test	Performing respiratory work before an exercise test affects the oxygenation of the legs and respiratory muscles but not the frontal cortex.
Radel et al. (2017)	Ergometer cycle	-	The participants performed 10 min or 60 min exercising	2-channel NIRS	22 (15M), 21.27 ± 2.07	Attentional focus as assessed three times during performing exercise by indicating a point in an analog range of completely on task to completely off task.	Based on oxyHb in right dorsolateral PFC and right medial frontal cortex, the brain's region associated with mental effort is disengaged with the brain's region linked to resting activity in order to keep mental resources for the maintenance of exercise.
Racinais et al. (2014)	Ergometer cycle	Not below 70	-	2-channel NIRS and 4-channel EMG	25 cyclists, 37 ± 8 years	The workload was increased by 25 W/min until the cycling rate drops below 70	Metabolic and ventilatory events may affect both muscle and cerebral oxygenation levels, and in turn, muscle employment
Smith and Billaut (2010)	Ergometer cycle	Qualitat-ively: Low/ medium/ high	Ten set of 10-s cycling with 30 s of rest	2-channel NIRS and EMG electrodes	13M soccer and rugby players, 23.6 ± 3.7	Subjects were exposed to a gas for 10-min while sitting on the ergometer. The gases used in this experiment were: normoxia, and hypoxia	During repeated short sprint cycling, although O_2_ availability influences the PFC, it doesn't affect the muscles.
Shibuya et al. (2004)	Ergometer cycle	90 to find the maximal oxygen uptake	147.2 ± 3.4 s	NIRS	5M, 24.6 ± 0.4	Subjects breathed through mask connected to hot wire flow meter to measure the respiratory flow.	Exhaustive exercise induces the decrease of cerebral function. Fatigue resulting from dynamic exercise decreases the cerebral cortex activity.
Subudhi et al. (2007)	Ergometer cycle	Above 50	Until the participants get exhausted	2-channel NIRS	13M cyclist, 30 ± 7	Subjects inhaled gas for less than 2 min before the experiment. Gas: normoxic or hypoxic	NIRS study on athletes show that incremental exercise performance under normoxic conditions is not possible to be limited by changes in cerebral oxygenation.
Subudhi et al. (2009)	Ergometer cycle	-	Until the participants get exhausted	Multi-channel NIRS	25 (23M)	For the experiments two gases of normoxic and hypoxic were used	During high-intensity, cortical deoxygenation is not restricted to the brain's pre-frontal part. It is possible that deoxygenation in pre-motor and motor cortices contribute to fatigue and/or decision to stop exercising.
Thomas and Stephane (2008)	Ergometer cycle	Above 60	13.3 ± 0.3 min	2-channel NIRS and EMG	13M right-handed, 24.9 ± 1.5	All participants had 6.1 ± 0.9 h/week training	During progressive maximal cycling exercise, a reduction in PFC oxygenation before motor performance failure is reported.
Pires et al. (2016)	A speed bicycle attached to a cycle-simulator	Self-paced 4 km time trial (TT4km) and maximal control-pace incremental test (MIT) with 80	699 ± 67 s for MIT and 359 ± 17 s for TT4km	32-channel EEG and 32 × 32 NIRS and a pair of EMG and gas analyzer for recording Cardiopulmonary Data	9M trained road cyclists, 32.9 ± 7.3	7 min warm up was performed before the experiment. This includes 5-min TT4km and 2-min MIT at 80	According to oxyHb in PFC and vastus lateralis muscle, at the closing stage of different cycling task, when the oxygen level (VO_2*MAX*_) is matched, similar motor output (EMG and motor output) is recorded though existence of different disturbances before the final point. Activation of M1 in through the exercises may represent that this part plays a role in centrally-coordinated exercise regulation.
Fumoto et al. (2010)	Ergometer cycle	60	15 min	24-channel NIRS	10 (9M), 32 ± 2.2	To assess psychological mood, subjects were asked to answer questionnaires.	Cycling task increases the brain's activity in ventral PFC region. This may cause a reduction in negative mood.
Ludyga et al. (2016)	Ergometer cycle	60, 90, and 120	3-min for each speed	32-channel EEG	36 (24M) cyclists 27 ± 3	The participants had at least 4 h cycling training per week within the last 6 months before the experiment.	Improvement in cycling training is closely related to brain cortical activity. Also, the higher cadence, the greater brain functional response.
Jain et al. (2013)	Stationary bicycle with a rigid, reclined backboard	2.1 s/cycle (±0.5 s/cycle)	20 min with a short break after 10 min	EEG: 64 channels EMG: 10 Channels	10, Range: 22-32 Median: 26	A warm-up consisting of 5-min self-paced walking and 2-min cycling at 100 W with pedal cadence of 80 rpm was performed	During pedaling, the brain processes a great amount of sensory activities. Cortical activities in pedaling reaches to its maximum in transitioning the legs from flexion to extension and vice versa
Schneider et al. (2013)	Ergometer cycle	Five pedaling exercises at 90	2	32-channel EEG and 7 muscle recording by EMG and electronically braked cycle ergometer	8, 5M aged 27 ± 4 and 3 female aged 24 ± 2	A standardized warm-up (i.e., 5 min at 1 W/kg, 2 min at 3 W/kg, and 1 min at 5 W/kg) and a 5-min recovery period was used before doing the task.	Besides showing the possibility of localizing brain cortical activity during pedaling activity, it is shown that motor cortex activity increases with the increasing of power level and significantly mirrored muscle activity.
Enders et al. (2016)	Ergometer cycle	97 by average (between 90 and 100)	7:04 min by average (range: 6:01 to 8:58 min)	64-channel EEG cap EMG	10M experienced cyclists	Each subject performed the tests on 3 different days. First day: finding the maximum aerobic power of participants Second day: perfuming the test at 85% of individual ability and familiarization. Third day: repeating the second day test and data recording	By increasing the fatigue, EEG power increases. The maximum increase occurs in frontal area of the cortex. timing of event-related desynchronization occurring in SMA denotes the source of producing force and its transition from flexion to extension in pedaling.
Fontes et al. (2020)	Ergometer cycle	60	30 s cycling and 30 s rest for four times	fMRI	22M, 24.4 ± 7.1	The intensity of the cycling was increased by 25 W at each round	By increasing the exercise's intensity, PFC activities decreases. Cerebellum was activated only in low-intense activity while motor cortex is activated in low and high intense activities.

### 3.4. Type 1-2: Walking-running

In this section, three research were found that have investigated both walking and running their studies, which meet the defined criteria for research selection in this review. These studies are investigated in Table 5.

**Table 5:** Brain-body imaging in walking-running.

**References**	**Surface**	**Dist. (m)**	**Speed Km/h**	**Duration (min)**	**Sensors**	**Participants No. and age**	**Special condition**	**Contribution**
Suzuki et al. (2004)	Treadmill	-	Walking: 3, 5, and running: 9	30 s rest, 90s locomotion, 30 s rest. Three repetition for each subject	NIRS (42 channel) with 28 optodes consisting of 12 light-source fibers and 16 detectors	9, right-handed, healthy subjects (7M 28.1 ± 7.4, Range 22–46	Starting task was selected randomly between 3 or 5 km/h speed. Participants could swing their hands freely.	In the frontal cortices, in contrast to deoxyHb, oxyHb increases in acceleration period proceeded by locomotion task. This change in oxygenated hemoglobin is greater in PFC and M1 at high-speed locomotion and there is less change in SMC. Consequently, to adapt to locomotor speed, PFC and M1 play crucial roles.
Nordin et al. (2019b)	Treadmill	-	Walking: 1.8, 3.6, 5.4, 7.2 and running 7.2, 9	18 min (3 min for each speed)	128-channel EEG, 8-channel EMG, optitrack with 10 cameras	9 (6M)	random obstacles were added to during walking/running on the treadmill	The dual-layer EEG cap reduced the artifacts effects on the data. Spectral power of delta, theta, and alpha frequency bands in SMA and PMC increased within 200 ms after the obstacle presence.
Jahn et al. (2004)	Imaginary	-	Walking: 3.6 and running: 9	0.33	fMRI (34 slices of brain was covered)	13, mean: 27.3 range: 21–35	Imaginary walking with closed eyes in supine condition: tasks: rest, standing, walking, and running in 20-s sequences	In slow walking spatial navigation plays a more significant role and this role is played by the parahippocampal cortex. In an unhindered locomotion such as running vestibular and somatosensory cortex get deactivated and this prevents the disruptive effect on the spinal pattern and sensory signals.

### 3.5. Type 1–2: Walking-cycling

Only one paper shown in Table 6 was found that has examined both walking and cycling.

**Table 6:** Brain-body imaging in walking-cycling.

**References**	**Surface**	**Dist. (m)**	**Speed Km/h**	**Duration (min)**	**Sensors**	**Participants No. and age**	**Special condition**	**Contribution**
Storzer et al. (2016)	OG/ Ergometer bicycle	-	40 strides per min (41.5 ± 2.88) for walking and 40 rpm: (40.9 ± 1.72) for bicycling	10 s movement (bicycling or walking) then 10 s rest for 50 times. Then, continues 2 min movement.	18-channel EEG and 6-channel EMG	14 (8M), 24.9 ± 3.0	The speed adjustment is based on prior instruction.	cortical activation during bicycling and walking are compared. During movement, while bicycling is associated with stronger decrease in beta power, walking is associated with alpha power reduction.

### 3.6. Type 4: Dual walking task-spontaneous locomotive and cognitive demands

By switching from single tasks to dual tasks, the age-related gait changes are more distinguished (Beurskens and Bock, 2012) the reason roots in the fact that the cognitive resources should compensate for the motor impairments (Mirelman et al., 2017). Because of this reason, in this type of locomotion task, usually both young adults (YA) and old adults (OA) are examined. The Dual task walking studies are presented in Table 7.

**Table 7:** Brain-body imaging in dual-task walking.

**References**	**Surface**	**Dist. (m)**	**Speed Km/h**	**Duration (min)**	**Sensors**	**Participants No. and age**	**Special condition**	**Contribution**
Mirelman et al. (2017)	OG/ Mat	30	SW-YA: 1.349 ± 0.157 SW-OA: 1.069 ± 0.1137 DTW-YA: 1.238 ± 0.136 DTW-OA: 1.058 ± 0.123	-	fNIRS (6 channel), walkway gait pressure mat	YA: 23 (10M): 30.9 ± 3.7 and OA: 20 (10M): 69.7 ± 5.8	Each round started and ended with 20 s of standing quietly, with the instruction to refrain from talking and moving the head. More complex walking: negotiating with two physical obstacles during walking. DTW: walking while talking (subtracting 3 s from a 3 digit, predefined number)	Needed cognition increases for both young and older people but for older people is more significant. Gait variability in older adults increases with the increase in pre-frontal activation. pre-frontal activation in older people is higher indicating older people rely more on their cognitive resources during walking. Neural activation in the PFC increases with task complexity, similarly, in both younger and older adults.
Holtzer et al. (2011)	OG/ Room	4.572	SW-YA: 0.1222 ± 0.175 SW-Old 0.716 ± 0.177 DTW-YA: 0.810 ± 0.175 DTW-OA: 0.362 ± 0.131	-	fNIRS (16 channels)	YA: 11 (4M): range: 19–29 OA: 11 (4M): range: 69–88	quiet room wearing comfortable footwear with the fNIRS attached to the front of the head. DTW: walking while talking alternate letters of the alphabet	PFC activation increases in WWT compared to mere walking. This increase is higher in young adults than older adults (contradiction with Mirelman et al., 2017). The results are compared to the WWT and shown that single walking task needs less cognitive resources.
Lu et al. (2015)	OG	-	Self-paced walking	1 min	fNIRS 8 × 8	17 (9M), 23.1 ± 1.5	WCT: subtracting 7 from a 3-digit number and speaking out the number. WMT: carrying a 600-ml bottle on a tray while walking	PFC, M1, and supplemental motor areas data obtained by fNIRS show that left-PFC has the highest oxyHb during WCT and there is a minor increase in oxyHb in initial phases of NW and WMT. M1 and supplementary areas get more activated during WCT and WMT. WCT cause a reduction in cadence, stride time, and stride length while WMT only diminishes the stride length.
Makizako et al. (2013)	OG	-	Self-paced walking (SW: 3.5 ± 0.6, DTW: 3.1 ± 0.7)	20 s for performing a SW or DTW	16-channel fNIRS (6 emitters and 6 detectors)	16 right-handed OA (10M) with 65 years or older	Each participant completed three SW and DTW. 10 s rest before the task and 20 s rest after the task were considered.	During DTW, pre-frontal activation is observed among older adults with mild cognitive impairment
Mirelman et al. (2014)	OG/ 7-meter sensor-carpet	5 walks of 30 meters for each trial	SW (4.86 ± 0.36), walking while counting (4.64 ± 0.54), walking while S7 (4.43 ± 0.50)	20 s during the task	6-channel NIRS	23 (10M), 30.9 ± 3.7	The conditions are: 1-SW 2-walking while counting forward 3- walking while subtracting 7 from a 3-digit number (S7) 4-standing while S7	It is shown that DTW is coupled with frontal brain activation. The observed changes are directly related to the cognition during walking and not verbalization.
Talamonti et al. (2021)	OG	-	SW: 4.169 ± 0.130 DT: 3.859 ± 0.144	5 min combination 30 s walking/ cognition task/ dual task walking	256-channel fNIRS	24, Older than 60, Participants were divided to two groups of high and low cardiovascular risk factors (HCVRFs and LCRFS) based on Framingham score.	Cognitive task: remembering 2-back heard number. Dual task walking: performing cognitive task while walking	HCVRFs show greater task-related cortical response specifically in pre-frontal caudal and rostral dorsal regions in the beginning of 12-month training. Physical training had more cortical activation reduction for HCVRFs. Cognitive performance and stable gait speed throughout are associated with 12-month physical training.
Pizzamiglio et al. (2017)	OG	-	Self-paced walking, walking while conversing with a friend and lastly walking while texting with a smartphone.	-	64-EEG channel, 2 digital force sensing resistor sensors for recording movement. A digital button (1-to-0 active edge) to distinguish start and end points	14 (5M), 26 ± 3	3 min of resting standing still (i.e., baseline) with their eyes open looking at a standard spot on a blank wall. Then walking for the purpose of familiarization and then recording for mere walking, then walking while conversing, finally walking while texting	Real-life activities are associated with different frequency-specific neural biomarkers. Walking while conversing is integrated with an increase of theta and beta neural power in electrodes located over left-frontal and right parietal regions. However, walking while texting is accompanied with a decrease of β neural power in a cluster of electrodes over the frontal-M1 and SMC.
de Tommaso et al. (2015)	OG	10	Self-paced	15 min	21-channel EEG, 4-channel EMG	17 (5M), Range: 18-65	Each subject did: sitting (5 min), standing (5 min), walking (5 min), P300 oddball was performed during standing and walking	P300 component amplitude increases during walking compared to standing. There is a negative correlation between age and P300 component vanishing during walking. According to motor-cortex and EMG activities, abnormal gait is distinguished from normal ones.
Meester et al. (2014)	Treadmill	-	SW: 4.39 ± 0.86 faster walking: 5.33 ± 0.94	30 s for performing the task and 20–40 s for rest. For five times at two speeds	4-channel fNIRS with two sources and two detectors and spinal cord reflex activity measured by soleus H-reflex	17 (7M), 15 right-handed and 2 left-handed. 27.8 ± 6.3, Range: 22–44	The cognitive task was counting backward in steps of seven from a defined number.	Although PFC activation doesn't change by increasing the walking speed, it would be activated in response to cognitive loads.
Eggenberger et al. (2016)	Treadmill	-	0.2, 3, 5 for walking	9 min walk 30 min exergame	fNIRS (2 sensors)	19, 74.9 ± 6.9 for exergame, 14, 74.9 ± 6.9 for balance	intermittent Interventions of exergame and balance during walking	Intermittent of exergame and balance reduce the oxygenation of the PFC. This reduction is more significant in exergame. This reduction could be relevant to improve mobility and falls prevention in the elderly.
Fraser et al. (2016)	Treadmill	-	YA: 2.64 OA: 1.78 And also preferred speed	2 min	fNIRS (16 detector)	19 YA 21.83 ± 1.92 14 OA: 66.85 ± 5.26	DTW: Walking and talking and remembering words and report immediately (1-back or 2-back)	After controlling the walking speed, the difference between YA and OA could be revealed as when the difficulty of task was increased, oxyHb in PFC of OA was increased.
Lau et al. (2014)	Treadmill	-	2.88 and 4.5	5 min standing and 10 min walking	248-channels EEG	8 (7M), Range: 20-31	Task: visual oddball discrimination, 20% target and 80% standard stimuli were displayed on a monitor to the participants at eye level 1 m in front of them.	Walking has lower functional connectivity between SMC areas than standing.
Castermans et al. (2011)	Treadmill	-	1.5, 3, and 4.5	6.5 min	32-channel EEG	7, Range: 25–33	The dual task was counting the green letters appearing during 3 s on a screen in front of the participants. Task: 0.2 s flashing light, 0.1 s between two flashes and 1 s interval and repeating that for 12 times for 25 target letters.	Feasibility of suing P300 during walking while recording EEG signals from parietal and occipital areas is shown, which can be beneficial for ambulatory conditions.
Malcolm et al. (2015)	Treadmill	-	YA: 2.4 and 5 OA: 2.4 to 4.8 (3.5 by average)	About 4 min for a block	EEG (72 channel)	17 YA (9M), 27.2 ± 4.6 Range: 21.8–36.1, 16 OA (7M), 63.9 ± 4.0, Range: 57.7- 71.0	OA performed five blocks of the response inhibition task while sitting, 9 or 10 blocks while walking and two blocks only walking. YA completed three or four blocks sitting, a minimum of four blocks walking slowly (range: 4-8 blocks), at least four blocks walking quickly (range: 4-8 blocks) and two blocks of each speed walking without the task. Task: speeded visual Go/No-Go task	By examining the variability and time of stride in different configurations, only the OA's accuracy drops significantly when performing inhibitory task while walking. Also, the brain's performance in YA is more modulated than OA according to the EEG data of cortical activities. The reason might be an age-associated loss in flexible resource allocation across multiple tasks.
De Sanctis et al. (2014)	Treadmill	-	2.4, 5	About 4 min for a block	EEG (72 channel)	18 (10M) Range: 21.8-36.1, Mean: 27.2	Doing a Go/No-Go task by shown pictures and selecting by mouse while sitting, walking slowly and walking briskly	When walking while doing another task, stride time in walking grows by increasing the cognition load of the task. Also, by increasing the age, the cortical motor behavior shifts from automatic to more controlled process.
Mazurek et al. (2021)	Treadmill	-	2.4, 5	About 4 min for a block	EEG (64 channels and 128 channels)	10, Range: 20–72	Doing Go/No-Go task while walking similar to De Sanctis et al. (2014) 16 blocks: one training block at the beginning, seven sitting blocks, seven walking blocks, and one task-free block (walking on the treadmill without a task)	Developing and easy customizing method for configuration EEG electrodes, which is improving the spatial localization of without specialized hardware or software.
Lau et al. (2012)	Treadmill	-	2.88	5 min standing followed by 10 min walking	248-channel EEG	8, Range: 20–31	Showing stimulus while the participants stand/walk should press a button when seeing the target stimuli.	Based on studying visual cortex in visual oddball discrimination during standing and walking, Weighted Phase Lag Index introduced as a potential method for recovering cognitive brain dynamics in the presence of gait-related artifacts.
Gramann et al. (2010)	Treadmill	-	2.88, 4.5	Two 10-min for each condition (60 min in total)	248-Channel EEG	11 (10M), 24.2 ± 3.4	A computer screen 50cm away from participants showed 80% non-target and 20% target stimuli, vertical or 45° rotated black cross for 500 ms. Three conditions were standing, slow walking, fast walking, and running (removed because of artifacts)	Inside or near right-lateral occipital cortex, and superior and inferior parietal cortex, according to ICA, are activated in the target -stimulus ERPs. In contrast, 40% of variance in 350–500 ms of the target-response ERPs was accounted for all movement conditions with activation in or near anterior cingulate cortex.

## 4. Results

### 4.1. Task analysis

**Figure 3:**
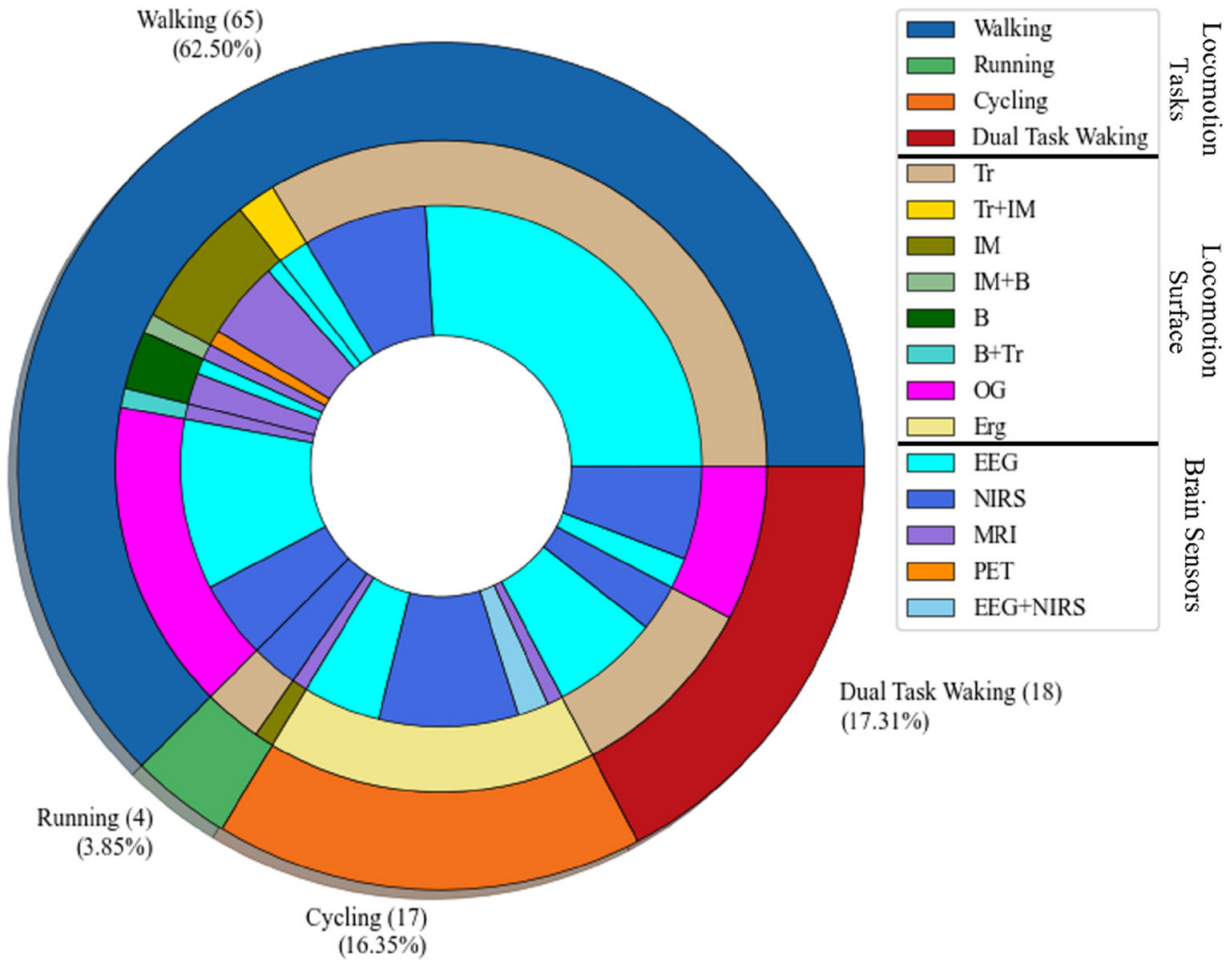
Task-Surface-Brain sensor visualization: the most outer layer shows the type of the locomotion task; walking, running, cycling, and dual-task walking. The middle layer shows the type of the surface used for the body-brain imaging experiment, which are Tr: treadmill, IM: imaginary task, B: Board with pedals, OG: overground, Erg: Ergometer cycling. The most inner layer shows the type of the brain sensor used in the brain-body imaging.

The filtered papers based on the mentioned criteria are 102 papers. Most of the papers, 65, conducted research on low-intensity locomotion, walking, only 4 papers examined running, 17 papers examined cycling, and 18 papers conducted the experiment on dual task, which considers the locomotive and cognitive demands simultaneously. Figure 3 shows the visualization of the tasks, the surface that the experiment has been conducted on, and the brain sensor that has been employed for the experiment. In this regard, the most outer layer of the circle shows the investigated locomotion tasks (walking, running, cycling, and dual-task walking). The middle circle demonstrates the type of the surface (Tr: treadmill, IM: imaginary, OG: overground, Erg: Ergometer bicycle, B: on a board). The type of brain sensor used in the research are shown in the most inner circle (EEG, NIRS, MRI, and PET).

### 4.2. Analysis of locomotive intensity

**Figure 4:**
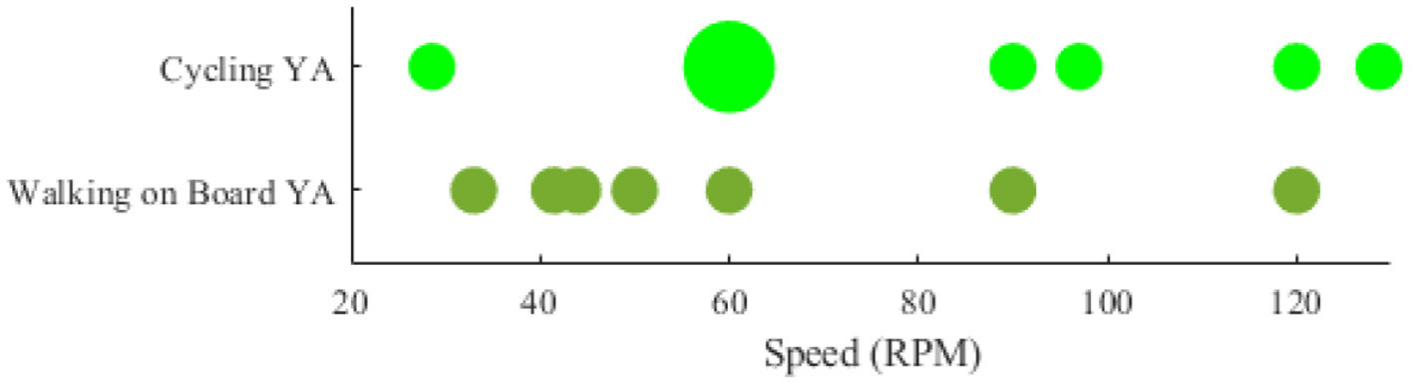
Comparing locomotive intensity in two types of brain-body imaging tasks performed with different rotational speeds: 1) cycling 2) walking on a board with pedals. Only younger adults have been employed to perform these tasks.

**Figure 5:**
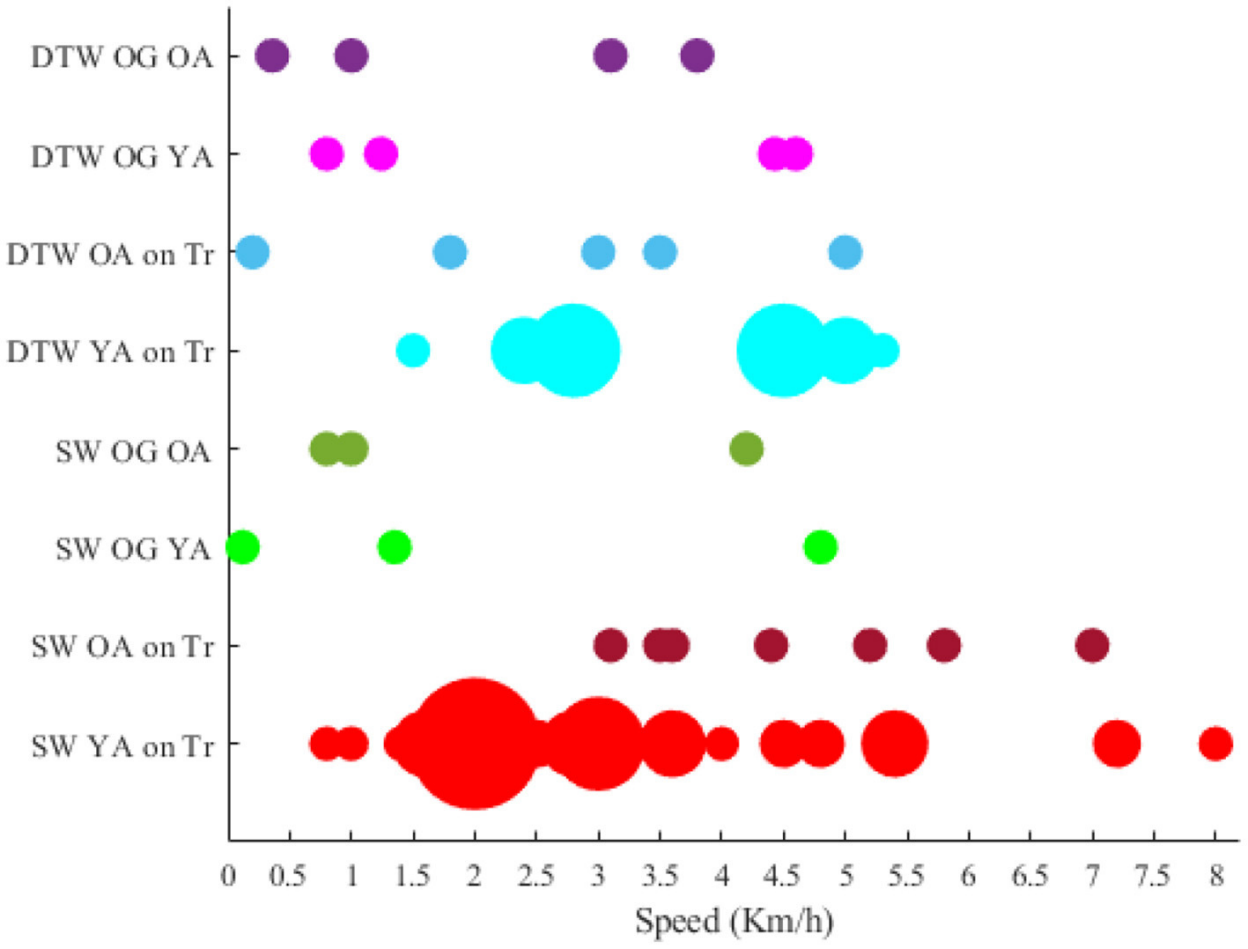
Intensity of locomotion in existing experiment protocols.

Among the researches that have declared the intensity (e.g., speed) that locomotion has been performed, the papers that have stated their speed (or an average of speed) have been compared with each other. However, the researches that the speed was not defined quantitatively and constant (e.g., the speed was dependent on the length of the participants' feet length Wagner et al., 2012, 2014; Seeber et al., 2014) were excluded from the visualization. Accordingly, the rotational velocities when the brain-body imaging experiment is using an ergometer or a pedaling board are visualized in Figure 4. The experiment using 60 rpm in cycling has been considered a reference speed for most researchers. Besides the rotational velocities of Figure 4, the linear speed for SW and DTW on different surfaces (i.e., Tr and OG) for YA and OA are shown in Figure 5. The bigger the bulb, the more repetition on reported speed. For example, walking at the speed of 2 Km/h is the main standard for researchers on the treadmill when the participants are YA. In addition, in each experiment surface, a trend of using a higher speed for YA compared to the speed used for OA can also be observed in the reviewed papers in this figure. For instance, in DTW OG, the maximum speed reported for YA is 4.6 Km/h, although the maximum speed reported for OA in DTW OG is about 3.8 Km/h.

### 4.3. Analysis of participants' age

**Figure 6:**
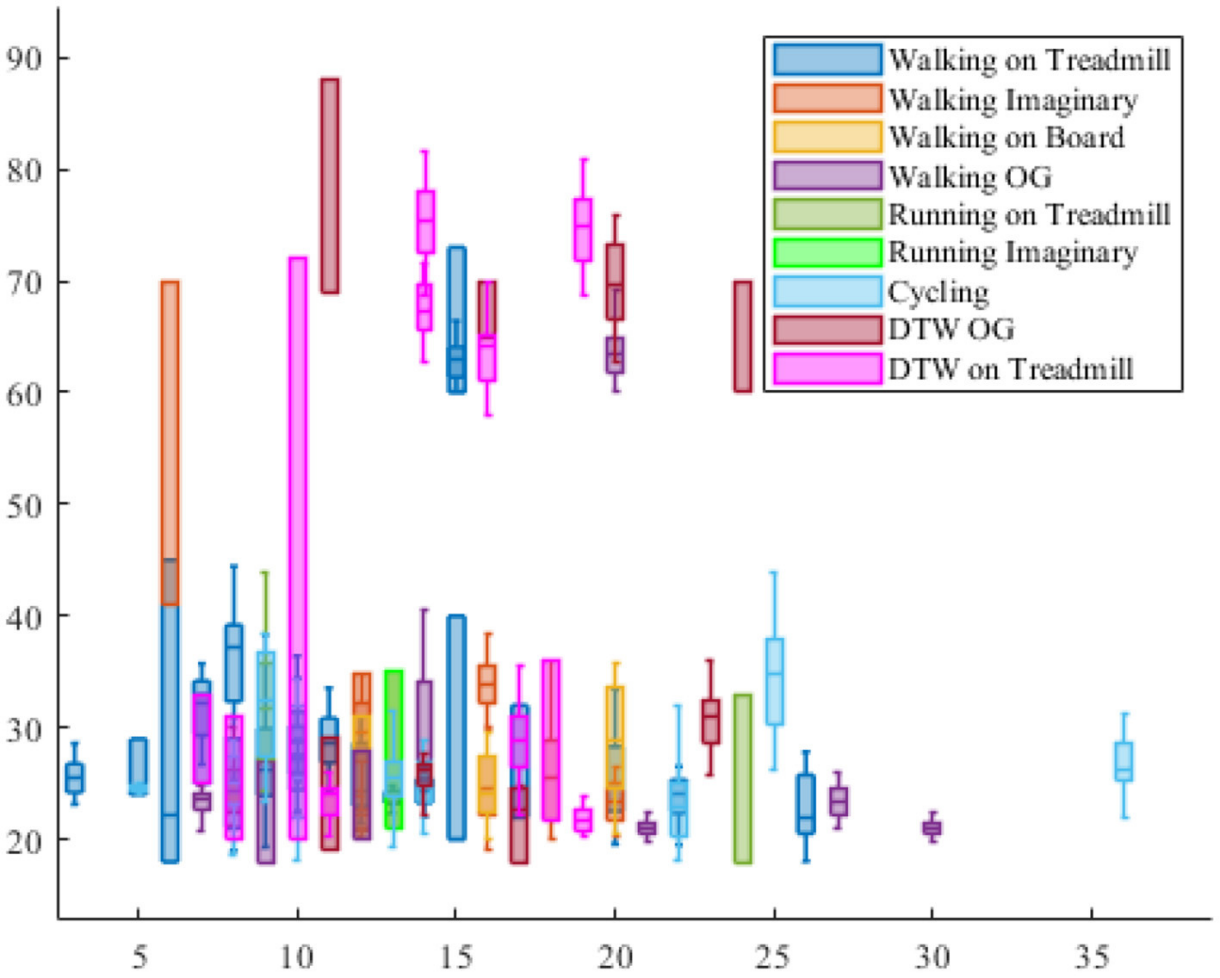
The participants' age distribution in each locomotion experiment. The vertical axis shows the age of the participants and the horizontal axis shows the number of participant in the reported experiment protocol.

To analyze the age of the participants, when the age of the participant is unknown, the research is excluded in the visualization of Figure 6. In this regard, there is a significant gap in the ages between 40 and 60 years old as only studies had participants in this range of age.

### 4.4. Analysis of locomotion duration

**Figure 7:**
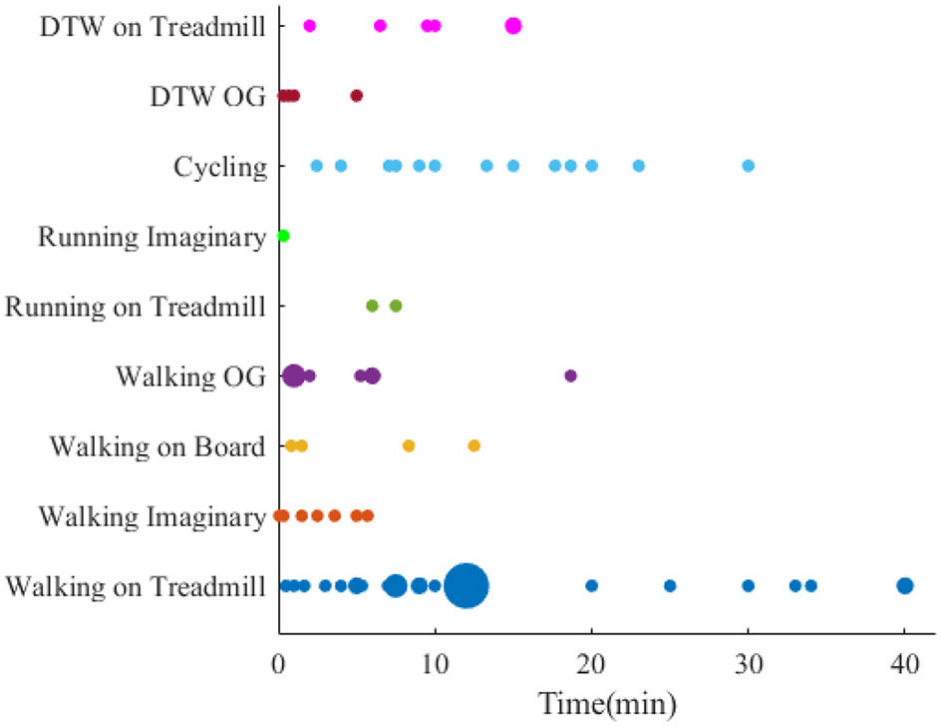
Brain-body imaging experiments' duration.

The duration of locomotion task varies from seconds to hours (Figure 7). In this regard, when it comes to single walking, the experiment on the treadmill with a duration of 12 min is the primary source for experiments. To make the results of studies with different duration comparable, the effect of fatigue on the muscles needs to be studied, and one solution could be recording the data after the locomotion task for a specific time.

### 4.5. Analysis of locomotion distance

**Figure 8:**
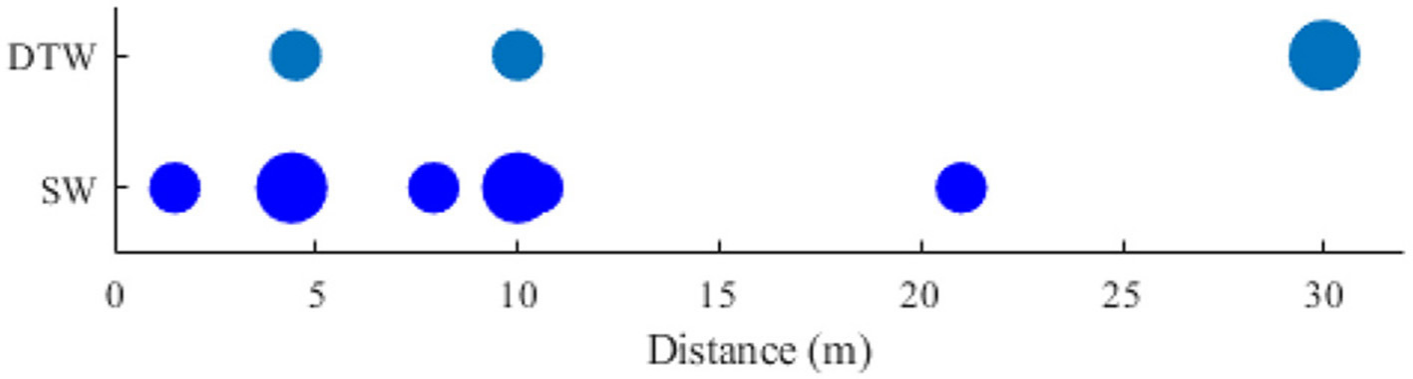
Comparing distance the walk distance in two types of Brain-body imaging experiments: 1) DTW and 2) SW.

Besides the duration, another parameter for assessing the locomotion demands is the distance when the participants were asked to walk overground. The walk distance falls in a range of 1 to 150 meters. For better demonstration, only the distances up to 30 meters are shown in Figure 8.

### 4.6. Brain activation in brain-body imaging experiments

**Figure 9:**
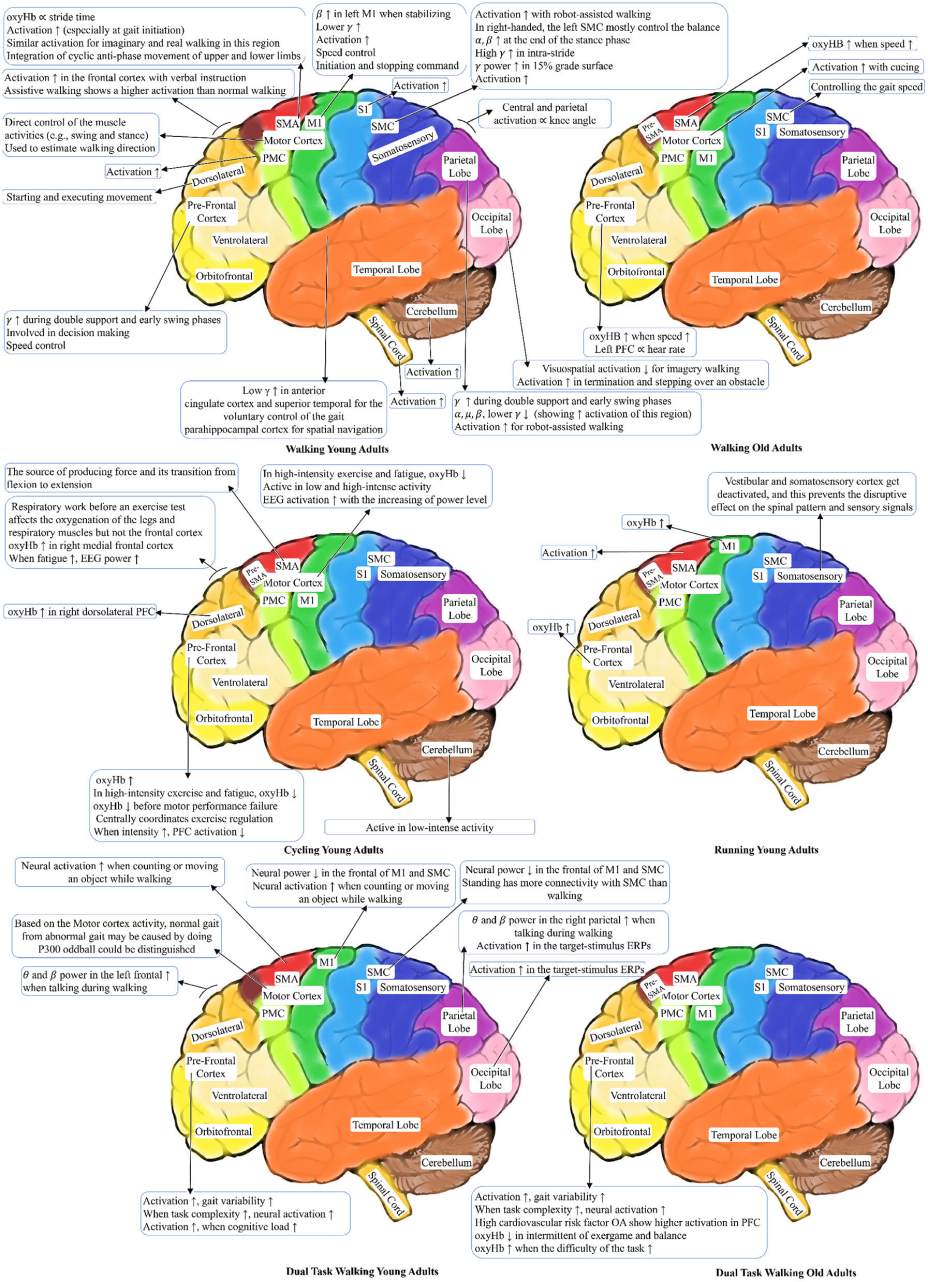
Brain areas activation patterns during locomotion tasks.

In this section, the corresponding brain activation to the locomotion is presented. Comparison of the brain activation areas for the locomotion tasks are shown in Figure 9. In this figure, we have only shown the results of the reviewed papers that have specifically mentioned the brain regions' activation sources and their effects. However, if a study generally describes the cortex area is excluded from this visualization. In this figure, ∝ is the proportional symbol, ↑ shows increasing, and ↓ shows reduction of an item. For instance, when the OA's walk speed increases, oxyHB in the Supplementary Motor Area increases.

## 5. Discussion

In this section, the configurations of locomotive and cognitive demands, the research interests regarding neurological and musculoskeletal drivers, and the observational constraints from the sensing techniques are discussed concerning the impact of brain-body imaging sensors on the design methodology of experimental protocols for measuring dynamics of brain, body, and behavior. Since few papers in the field have adopted a conceptual framework to evaluate the quality of the neurological and musculoskeletal correlates of human locomotion extracted using various methods, it is not easy to compare these state-of-the-art methods. Therefore, in this section, we focus on establishing the elements of the proposed conceptual framework and discuss how to assess the neurological and musculoskeletal measures extracted from brain-body imaging sensors for further clinical use.

### 5.1. Neurological and musculoskeletal drivers

According to our systematic review results, most studies followed the philosophy of medical diagnosis that is rooted in conducting statistical analysis between different groups (e.g., young vs. old, control vs. patient). These groups were asked to perform specific behavioral protocols under which their performances were supposed to show neurological and musculoskeletal differences reflected by the brain-body imaging data. Therefore, various experimental protocols were explored to capture the differences in statistical norms among groups (Kashuba et al., 2020; Warmerdam et al., 2020). The typical statistical analysis approaches include comparisons of statistical norms (e.g., *p*-value, effect size) and classifications based on machine learning (e.g., accuracy, precision) (Figueiredo et al., 2018; Hatami et al., 2019; Patil et al., 2019; Hausmann et al., 2021). Few studies adopted representation learning methods based on deep neural networks to explore patterns and features of brain and motion signals to show the group differences (Vásquez-Correa et al., 2018; Talo et al., 2019; Song et al., 2021). Furthermore, specific quantitative assessments, such as clinical outcomes or symptoms, were identified as ground truth so that the same reference could examine both groups. However, no consensus on experimental protocols caused challenges in determining reliable ground truth or references. Otherwise, few studies provided explanations and rationales for the design methodology of behavioral experiments.

Although it lacks standardization of experimental protocols, existing studies still generated consistent conclusions on neurological and musculoskeletal correlates of human locomotion. For example, most studies revealed that the active brain regions during various walking protocols include the motor, sensory, and prefrontal cortexes. The brain areas involved in walking behavior span SMA, premotor cortex, sensorimotor, M1, and left and right prefrontal cortex. Notably, the age-related changes showed that the involvement of PFC increased among old adults, especially during high-speed walking. When the subjects need to adjust their postures for cycling, the involvement of PFC decreases in order to keep mental resources for the maintenance of these additional requirements during exercise. The PFC has been implicated in planning complex cognitive behavior, especially in the resting status. Few studies explored the running protocols; therefore, there is little consistent knowledge regarding neurological and musculoskeletal correlates of human running. It is noteworthy that when subjects are required to conduct spontaneous cognitive tasks, the PFC activation increases in all age populations, and its increase is more significant in older adults with and without cognitive impairment. These conclusions have motivated many hypothesis-driven research projects on disease-related changes in neurological and musculoskeletal correlates of human locomotion.

Besides the consistent understanding of neurological correlates of human locomotion, mixed results and conclusions exist due to the heterogeneity of participants' neurological expressions, musculoskeletal variance under imprecise experimental protocols, and observation constraints by the sensing techniques. For instance, some studies concluded that the cerebral cortex controls multiple muscles hierarchically through a few synergies during walking. In contrast, few studies argued that the role of cortical control during walking might not be valid due to the motion artifacts of EEG. Furthermore. Subjects that conducted actual walking were more accurately classified than subjects with imaginary walking. Subjects that walked on a treadmill showed different band activities captured by EEG compared to subjects who walked over the ground. These inconsistent results require further investment in establishing protocols, standardization, and benchmarking tools, which also motivated this research work.

### 5.2. Locomotive and cognitive demands

The primary assumption underlying the experimental protocols is that the locomotive and cognitive demands could stimulate changes and patterns in neural activity, which the brain-body sensors could pick up (Ladouce et al., 2019). Thus, the knowledge of the simulation mechanisms further guides the treatment and rehabilitation strategies for potential clinical outcomes. However, most experimental protocols involve low-intensity locomotion tasks, such as walking and cycling, while few studies conducted high-intensity locomotion tasks, such as running. In addition, little research explained the design methodology of the experimental protocol, especially the research hypothesis of which types of neural activities might be expected under the experimental configurations, such as duration, intervals, and frequency. Also, conducting behavioral studies on human subjects is always challenging, even more, if considering psychological factors such as state of mind, concentration, and technical dexterity. Therefore, some studies integrated cognitive demands into locomotive tasks, such as avoiding obstacles, determining walking directions, or following an avatar. Other studies developed a dual-task paradigm to examine the involvement of cognition in human locomotion.

As illustrated in Figure 1, the conceptual framework argued that more work is needed to examine the simulation mechanisms, thus developing a better design methodology for experimental protocols. The systematic review results showed that several challenging questions remain in the research field. First, benchmarking the locomotive and cognitive demands will be needed. Locomotive tasks have several configurable parameters, such as duration, intervals, and frequency, which need to divide into several levels or intensities. Cognitive tasks also have configurable parameters, such as type and complexity, depending on working memory and executive functions. For instance, for a specific subpopulation's demographic, locomotion and cognition capacity, researchers in designing experimental protocol need a basic understanding of which levels of demands might stimulate the anticipated brain activity. Knowledge of locomotion disorder patterns and corresponding brain areas has helped establish experimental protocols for several neurological diseases, such as parkinsonian gait for Parkinson disease (Ghai et al., 2018) and NIH cognitive toolbox for cognitive impairment (Gershon et al., 2010). Second, manipulation of the tasks needs more research investment. Existing studies rarely consider how the sequential configuration of the tasks stimulates neural activity. Most protocols conducted a heuristic-designed sequence of locomotive tasks and anticipated the brain-body sensors could capture the subtle changes or patterns of neural activities. However, the loop from demands to brain and musculoskeletal activities to sensors, in Figure 1, shows that manipulation of the demands could generate richer information than the heuristic-designed protocol. Third, the experimental protocols should be easily administered to avoid confusion and distraction for participants. Few studies gave a clear description of how the protocols are being instructed. Cueing the participants toward specific tasks could have influenced the expected neural activity and cognitive performance. Therefore, most researches argued that when the experiment protocol is administered and instructed by assistance of a computer is less likely that neural activity and cognitive performance get adversely affected compared to the case that the experiment is controlled and instructed by interference of a human. Hence, replication of previous experiment protocols that were controlled by a human as examiner with a computer-assisted administration provides a high-quality data with removal of the human interference affects (Vrana and Vrana, 2017; Dror, 2020; Young et al., 2022). Moreover, computer assistance in the cognitive load assessment could make it feasible to conduct the experiment at places out of the clinics and laboratories. These places (e.g., participants' home or a local clinic) are accessible to the participants and individuals are comfortable to perform the experiment with assistance of a computer without interference of an examiner. As positive side effects of the computer-assisted experiment administration, high-quality and cost-effective patient care could be provided (Porrselvi, 2022; Young et al., 2022).

### 5.3. Observation constraints by the sensing techniques

Another dimension of our systematic review results is illustrating the impacts of the observation constraints by the brain-body sensing techniques. Previous work has reviewed and discussed the advantages and disadvantages of these techniques, including tolerance of motion artifacts and spatial and temporal resolutions. However, most studies reviewed in this work did not explain the rationale for selecting specific sensing techniques in experimental protocol design. According to our review results, it is obvious to see the impacts of sensing techniques on the research results and generated knowledge. Therefore, we summarize these impacts for enabling guidelines for future researchers to design experimental standards and benchmarks.

#### 5.3.1. Tolerance of motion artifacts

Prior knowledge of sensing techniques has concluded that among three of them, MRI has the lowest tolerance to motion artifacts, EEG less, and fNIRS the highest. Despite efforts in signal processing and denoising to improve each technique's tolerance to motion artifacts, most studies still followed the knowledge and showed different experimental results. Studies that adopted MRI has considered its limited tolerance of motion artifacts and mainly designed imaginary locomotion or simulated surrogate tasks rather than actual locomotion in their experiments (Stolbkov et al., 2019; Amemiya et al., 2021). The central assumption of these studies is that neurological correlates of these imaginary or surrogate tasks are closely related to neurological activation during actual locomotion. However, our summarized results in locomotion tables and Figure 9 showed that these imaginary or surrogate tasks could not simulate the complex dynamics the brain must execute to adjust and maintain the musculoskeletal patterns during actual locomotion. Especially studies that utilized EEG have shown that actual walking stimulated brain activation patterns that could be classified with higher precision than imagery walking. Therefore, studies that adopted EEG sensors designed a more comprehensive range of locomotion tasks, from imaginary walking and cycling to running. Furthermore, the fNIRS studies are preferred in high-intensity locomotion experiments. More than 50% of studies reviewed in this work that conducted cycling and running tasks adopted fNIRS sensors.

Sensitivity of EEG data to motion artifacts is a research concern and the results of some previous published research due to not considering an extensive removal of motion artifacts has been questioned. For instance, the reported EEG results indicating the changes in high-gamma frequency band during walking (Gwin et al., 2011) could be caused by motion artifacts as well (Castermans et al., 2014). To address the considerable drawback, different denoising methods to employ during or after data collection have been developed. During data collection, artifact removal is associated with hardware modifications. In this respect, one effective way has been introduced to separate electrophysiological signals from non-neural signals. To this end, in one approach, two layers are used below the EEG cap. A silicone layer is used on top of the scalp to block electrophysiological signal. Then, a simulated conductive scalp with similar impedance to human scalp is used to measure the voltage differences generated by gait dynamics (Snyder et al., 2015). In another approach, well-known as a EEG dual electrode design, simultaneously EEG data and isolated motion artifacts are recorded by pairs of the electrodes that are electrically independent and mechanically coupled (Nordin et al., 2018; Clark et al., 2020). After the data collection, denoising is coupled with software data processing. In this regard, Independent Component Analysis (ICA), low, high, and band band pass filter are routinely applied. Besides these remedies, ICA-based methods such as adaptive ICA mixture model algorithm (Palmer et al., 2006), extended infomax ICA (Lee et al., 1999) and multiple mixture ICA (Allen et al., 2000) approaches are utilized (Gwin et al., 2010). Moreover, a developed conductive head phantom and robotic motion platform has emerged as a powerful tool to analyze the artifact removal methods through generating a ground truth for EEG signal. This device is used to evaluate artifact removal methods such as dual-layer EEG and Artifact Subspace Reconstruction (Richer et al., 2020). Also, this device is used to show that electrodes with larger surface reduces the electrodes vulnerability to motion artifacts (Symeonidou et al., 2018). In addition, this device is employed to assess the effect of the motion artifacts parameters such as frequency and amplitude (Oliveira et al., 2016).

#### 5.3.2. Spatial and temporal resolutions

MRI has the highest spatial resolution among the three sensing techniques while fNIRS has the lowest (only centimeters under the skull) (Li et al., 2022b). Accordingly, MRI studies discussed their results with a detailed description of the brain cortex, such as SMA and the dorsal premotor cortex. Nonetheless, lacking the portability feature has significantly limited the application of the MRI in the brain-body imaging of locomotion studies mostly to imaginary task and thus, this scope of study has been deprived from the high spatial resolution of the MRI imaging technique. On the other hand, although EEG has lower spatial resolution than MRI, appropriate source localization approaches could improve its spatial resolution at a cortical level. When source localization is used, the spatial resolution of the fNIRS and EEG are comparable. However, EEG has the highest temporal resolution among the three methods and permits the highest and the most precise data brain investigation compared to the fNIRS and MRI methods. Thus, Looking into the results summarized in our work, we can see that studies that adopted EEG sensors provided the neurological and musculoskeletal correlates on a fine-grained temporal scale, such as neural activation patterns during different movement periods (e.g., swing, stance). These features has made the EEG the most popular technique in the explorations of the brain-body imaging of human locomotion.

#### 5.3.3. Miscellaneous constraints

We also observed other constraints in the design methodology of experimental protocols, such as imbalanced ages of participants. For example, most studies recruited participants aged below 40, while fewer recruited participants older than 60. A significant age gap (40–60) among the participants involved in these studies existed. However, the adults within this age range are baby boomers in the U.S., a large sector of the population, a group deemed by the Centers for Disease Control and Prevention (CDC) as mid-life stage adults. This age population is facing an increase in clinical and other preventive services to support maintaining good health into older age (Pearson-Stuttard et al., 2019; Doubeni et al., 2021), which means this age group should be a critical population for studies on neurological and musculoskeletal correlates of human locomotion. Although there might exist real-world challenges to recruiting this specific age population, additional investment in balancing the age distribution of the participants is needed to fulfill a cross-lifespan understanding of human locomotion and its neurological and musculoskeletal correlates.

## 6. Conclusion

This paper explores the topic of the design methodology of experimental protocols that aim to study neurological and musculoskeletal correlates of human locomotion using brain-body sensing techniques. The review of many types of neural activities stimulated by human locomotion demonstrates the importance of quantitative analysis using brain-body sensors in potential healthcare applications. By reviewing the current design methodology of experimental protocols, this paper illustrates that the protocol design significantly impacts the experimental results due to the heterogeneity of participants' neurological expressions, musculoskeletal variance under the imprecise locomotive and cognitive demands, and observation constraints by the sensing techniques. Finally, the impacts of the experimental protocols are discussed by reviewing the practical issues to provide implications and guidelines for future researchers to design experimental standards and benchmarks.

Brain-body imaging of human locomotion is a vast area of research. This paper focused on a significant research issue: how to reproduce human locomotion experiments. Therefore, we conducted a systematic review of existing experiment protocols to examine various settings and conditions, such as locomotion intensity, locomotion duration, locomotion distance, brain sensing technologies, and corresponding brain activation expressions. In future work, technologies for locomotion sensing and their advantages and disadvantages will be further examined and discussed. Also, upper-limb locomotion, such as shoulder, elbow, wrist, and finger movement, is a broad study that will be examined in another work. Finally, as the participants of this systematic review are healthy, similar brain-body imaging experiment exploration for neuromechanical disorders would be a valuable work to extend.

## Data availability statement

The original contributions presented in the study are included in the article/supplementary material, further inquiries can be directed to the corresponding author.

## Author contributions

JG designed the study, created the research question, and finalized the manuscript. SK implemented the study process, collected and analyzed the data, and initiated the manuscript. NJ developed the study motivation and significance, guided the data analysis, and revised the manuscript. All authors contributed to the article and approved the submitted version.
